# *Asparagopsis taxiformis* mitigates ruminant methane emissions via microbial modulation and inhibition of methyl-coenzyme M reductase

**DOI:** 10.3389/fmicb.2025.1586456

**Published:** 2025-04-25

**Authors:** Shuai Li, Yi Sun, Siguang Cao, Tongjun Guo, Xiong Tong, Zhifei Zhang, Jiajie Sun, Yufeng Yang, Qing Wang, Dagang Li, Li Min

**Affiliations:** ^1^Southern Marine Science and Engineering Guangdong Laboratory (Zhuhai), Ministry of Agriculture Key Laboratory of Animal Nutrition and Feed Science in South China, Institute of Animal Science, Guangdong Academy of Agricultural Sciences, Guangzhou, China; ^2^Guangdong Provincial Key Laboratory of Animal Nutrition Regulation, College of Animal Science, South China Agricultural University, Guangzhou, China; ^3^Southern Marine Science and Engineering Guangdong Laboratory (Zhuhai), Department of Ecology, Jinan University, Guangzhou, China; ^4^Key Laboratory of Xinjiang Feed Biotechnology, Feed Research Institute, Xinjiang Academy of Animal Science, Ürümqi, China; ^5^Agri-Food and Biosciences Institute, Hillsborough, United Kingdom

**Keywords:** methane synthesis, microbial regulation, low carbon, ruminant, methane inhibitor, *Asparagopsis taxiformis*

## Abstract

**Introduction:**

*Asparagopsis taxiformis* (*A. taxiformis*) has shown great potential to mitigate methane (CH_4_) emissions in recent years. This study aims to evaluate the impact of *A. taxiformis* on methane emissions and to fill the knowledge gap regarding its mechanisms of action in affecting CH_4_ metabolism and rumen fermentation.

**Methods:**

The experimental design consisted of a control group (CON) and test groups supplemented with 2% (Low), 5% (Mid), and 10% (High) of dried and freeze-dried treatment *A. taxiformis*, respectively, for 48 h of *in vitro* rumen fermentation. The optimal combination strategy for mitigating CH_4_ emissions was confirmed by analyzing nutrient degradation, CH_4_ production and rumen fermentation parameters, and the mechanism of action was analyzed by metagenomic and metabolomic approaches.

**Results and discussion:**

The results showed that freeze-dried treatment had better potential to mitigate CH_4_ emissions than dried treatment, and supplementation of freeze-dried treatments at Low, Mid, and High groups significantly reduced CH_4_ production by 32.44%, 98.53%, and 99.33%, respectively. However, the High group exhibited a huge negative impact on rumen fermentation. Therefore, subsequent analyses focused on the Low and Mid groups to explore the underlying mechanisms. Metagenomics analyses showed that supplementation of freeze-dried treatment with the Mid-level supplementation significantly increased the relative abundance of propionate-producing bacteria such as *Prevotella*, *Ruminobacter*, and *Succinivibrio*, while inhibited acetate-producing bacteria such as *Ruminococcus*, altered the pattern of volatile fatty acid (VFA) synthesis in the rumen, and reduced H_2_ availability for methanogenesis and promoted propionate production, indirectly alleviating CH_4_ production. Moreover, by suppressing the relative abundance of *Methanobrevibacter*, CH_4_ production in the rumen was directly suppressed. Furthermore, KEGG pathway analysis showed that *A. taxiformis* significantly inhibited the abundance of K00399, methyl-coenzyme M reductase alpha subunit, which directly inhibited CH_4_ synthesis. Metabolomics analysis of *A. taxiformis* supplementation significantly enriched ketoglutarate, malate, isocitrate, and melatonin, which may have reduced the release of rumen fermented H_2_, thereby mitigating CH_4_ emissions. In summary, freeze-dried treatment *A. taxiformis* at the 5% supplementation level achieved the optimal balance between CH_4_ mitigation and rumen fermentation efficiency.

## 1 Introduction

The agricultural sector is a major source of CH_4_ emissions, with livestock production contributing 37% of global CH_4_ emissions (Sun et al., 2023). Ruminal fermentation in ruminants is livestock’s primary source of CH_4_ emissions, resulting in a 2%–12% loss of total energy intake (Johnson and Johnson, 1995). Among all anthropogenic CH_4_ emissions, ruminant fermentation accounts for approximately 33%, making it the second largest source after fossil fuel activities (Crippa et al., 2021), while CH_4_ is 28 times more thermogenic than carbon dioxide (CO_2_). Therefore, effective CH_4_ mitigation strategies are crucial to reducing environmental hazards and enhancing the growth performance of ruminants. The rumen of ruminants is a complex ecosystem in which bacteria facilitate carbohydrate decomposition, and methanogenic archaea collaborate with these bacteria to produce CH_4_ by utilizing H_2_ generated through bacterial fermentation (Lyu and Liu, 2018). Dietary modulation of rumen microbiota represents the most viable strategy for CH_4_ mitigation, with feed additives demonstrating particular promise (Patra et al., 2017). Among these, chemical inhibitors have shown marked efficacy: 3-Nitrooxypropanol (3-NOP) reduces emissions by 20%–60% through competitive inhibition of methyl-coenzyme M reductase (MCR) (Jayanegara et al., 2018), while melatonin (10^−3^ M) achieves 50% reduction by decreasing *Methanobacterium* abundance and protozoa number (Fu et al., 2023). Natural additives, particularly seaweeds, exhibit comparable potential. The brown seaweed *Sargassum mcclurei* (2% DM) by decreasing *Methanobacterium* abundance and increasing propionate-producing bacteria, leading to an 18.85% CH_4_ reduction (Li et al., 2024).

Intriguingly, recent studies have demonstrated that the red seaweed *A. taxiformis* exhibits a potent inhibitory effect on CH_4_ emissions, capable of mitigating over 95% of CH_4_ when added to the diets (under both *in vivo* and *in vitro* experimental conditions) (Machado et al., 2016b; Kinley et al., 2020; Min et al., 2021; Glasson et al., 2022). The *A. taxiformis* is rich in halomethanes, haloalkanes, haloketones and haloacids, of which bromoform (CHBr_3_) is considered to be the most abundant (Woolard et al., 1979). These active ingredients confer on *A. taxiformis* the ability to efficiently inhibit CH_4_ by affecting the abundance of methanogenic archaea and by reacting with reduced vitamin B12 to produce an enzymatic inhibition that limits the rate of the methyltransferase step in CH_4_ synthesis (Wood et al., 1968). The efficacy of *A. taxiformis* in mitigating rumen CH_4_ emissions is significantly influenced by both supplementation levels (Machado et al., 2016b; Makkar et al., 2016), and treatment methods. Treatments at different temperatures (freeze-dried and dried) significantly affected the volatilization of methane-inhibiting haloalkanes and the cellular integrity of *A. taxiformis* (Vucko et al., 2017). Currently, freeze-dried treatment *A. taxiformis* is widely used as a CH_4_ inhibitor (Kinley et al., 2016b; Machado et al., 2016b; Kinley et al., 2020). However, considering the added cost of the application, it is also necessary to evaluate the CH_4_ emission mitigation effect of dried treatment (more convenience and low cost) of *A. taxiformis*.

We hypothesized that different treatments and supplementation levels may have different inhibitory effects on CH_4_ due to different levels of retention and content of bioactive compounds.

Therefore, the objectives of this study were to evaluate the effect of different treatments and supplementation levels of *A. taxiformis* on mitigating CH_4_ emissions and to assess the effect on rumen fermentation by analyzing *in vitro* rumen fermentation gas production, nutrient degradation, and rumen fermentation parameters. The mechanism of *A. taxiformis* in inhibiting rumen CH_4_ production was explored using metagenomics and metabolomics approaches.

## 2 Materials and methods

### 2.1 Animal experiment permit

This study was conducted in August 2023 at the Institute of Animal Science, Guangdong Academy of Agricultural Sciences, Guangzhou, China. Three Holstein cows were used in the experiment. The animal experiment protocol complied with the requirements of experimental animal welfare and ethics, as well as the regulations of the Ministry of Science and Technology of China. It was performed following the European Directive 2010/63/EU and S.I. No. 543 of 2012.

### 2.2 Collection and preparation of *Asparagopsis taxiformis*

We focused on *A. taxiformis* because it is a characteristic species of Guangdong coastal ecosystems, and more importantly, it exhibits exceptional performance in mitigating CH_4_ emissions. Accordingly, in this experiment, the red seaweed *A. taxiformis* was used as a supplement to mitigate ruminal CH_4_ emissions. The seaweed was harvested from Naozhou Island (Zhanjiang, Guangdong, China, 20°54′–21°10′N, 109°00–109°15′E). *Asparagopsis taxiformis* was submerged in fresh water for 1 min and then dried with a roller. The dried seaweed was divided into two parts, with one part subjected to conventional dried and the other to freeze-dried. Samples were dried at 65°C for 48 h as a dried treatment, and samples were frozen at −80°C overnight, then freeze-dried by Christ ALPHA2-4LSC (Chirst, Germany) at −50°C for 48 h under 0.1 mbar vacuum. Both treatments resulted in the seaweed being milled to a 1 mm particle size (Machado et al., 2018) and stored at −20°C until use in subsequent experiments.

### 2.3 Experimental design and treatment

#### 2.3.1 Seaweed bioactive components analysis

The determination of bioactive compounds in *A. taxiformis* samples was performed by Boende Testing Center (Qingdao, China). For FT-IR characterization, samples were homogenized with potassium bromide (1:100 w/w) and pressed into pellets and analyzed using a NiColet iS5 (Thermo Scientific, United States). For HPLC analysis, 200 mg of each sample were subjected to methanol extraction (20 mL, 10 min ultrasonication), filtered through 0.45 μm membranes, and analyzed using an Agilent 1260 (Agilent, United States) equipped with an SB-C18 column (250 mm × 4.6 mm, 5 μm). Subsequent LC-MS analysis was conducted on a Thermo TRACE 1610 (Thermo Scientific, United States) with a TG-5SILMS column (30 m × 0.25 mm × 0.25 μm), employing EI ionization at 1.0 mL/min flow rate. The mobile phase consisted of (A) 0.1% formic acid with 5 mM ammonium acetate and (B) methanol: acetonitrile (1:1 v/v), with detection in SCAN mode.

#### 2.3.2 *In vitro* rumen fermentation system

On the day of the experiment, to minimize individual variation, we selected three healthy Holstein cows with similar age (2.8 ± 0.5 years) and body weight (510 ± 25 kg) that were fed the same diet. The rumen fluid was collected 2 h after morning feeding. Rumen fluid samples were from donor animals using a stomach tube-based rumen fluid sampler (Anscitech, China). The sampler was orally inserted through the esophagus into the rumen following proper restraint. Approximately 200 mL of rumen fluid was manually aspirated from the rumen using a sterile syringe, with the first 50 mL of collected fluid being discarded to minimize salivary contamination. Between sampling procedures, the device was thoroughly rinsed with warm distilled water to prevent cross-contamination. The rumen fluids collected from the three cows were pooled in equal volumes (1:1:1), filtered through four layers of sterile gauze. The mixed fluid was then stored in a CO_₂_ flushed thermos preheated to 39°C for immediate transport to the laboratory.

The rumen buffer solution was prepared according to the method described by Menke and Steingass (1988). The solution is consisted of 400 mL H₂O, 0.1 mL solution A (containing CaCl₂·2H₂O 13.2 g/L, MnCl₂·4H₂O 10.0 g/L, CoCl₂·6H₂O 1.0 g/L, and FeCl₃·6H₂O 8.0 g/L), 200 mL solution B (NaHCO₃ 39 g/L), and 200 mL solution C (Na₂HPO₄ 5.7 g/L, KH₂PO₄ 6.2 g/L, MgSO₄·7H₂O 0.6 g/L), 1 mL resazurin (0.1% w/v) and 40 mL reducing solution (95 mL H₂O, 4 mL 1 N NaOH, and 625 mg Na₂S·9H₂O).

The rumen fluid and buffer solution are mixed evenly in a ratio of 1:2 (25 mL:50 mL). The mixing process is always carried out under constant temperature water at 39°C and carbon dioxide flushing. This study used fermentation bottles (100 mL), each containing 500 mg of fermentation substrate (forage: concentrates = 60:40) (Choi et al., 2022) and 75 mL of mixed rumen fluid. The fermentation substrate consisted of corn straw and concentrate supplementation. Both components were dried, milled to 1 mm, and stored in a desiccator until use in subsequent experiments. Different treatments of *A. taxiformis* (DM) were supplemented at 2, 5, and 10% (Low, Mid, and High) of the fermentation substrate DM. The control group (CON) without supplemented with *A. taxiformis*. Each supplementation level had 6 replicates, totaling 42 fermentation bottles, the experiment was repeated twice and 6 samples were randomly selected for subsequent experimental analysis. The bottle is filled with carbon dioxide and sealed with a butyl stopper and an aluminum cap to ensure the anaerobic environment required for fermentation. This study of *in vitro* rumen fermentation was carried out for 48 h in a constant temperature water shaker at 39°C and 85 rpm. In this experiment, the nutrient composition of the fermentation substrate is shown in Table 1.

**Table 1:** Chemical composition of substrates and *Asparagopsis taxiformis* used in the *in vitro* rumen fermentation (DM basis).

Parameter	Corn straw^1^	Concentrate^2^	*A. taxiformis*
DM %	26.26	93.53	NA
OM %	92.85	92.81	63.17
CP %	7.12	20.47	22.61
NDF %	38.49	16.61	38.39
ADF %	21.69	6.22	13.23
Ash %	7.14	7.18	36.83

DM, dry matter; OM, organic matter; CP, crude protein; NDF, neutral detergent fiber; ADF, acid detergent fiber; NA, not available. ^1^Corn stover harvested in experimental fields. ^2^Concentrate of composition: corn 500 g/kg, DDGS (Distiller’s dried grains with solubles) 235 g/kg, soybean meal 220 g/kg, stone powder 10 g/kg, dicalcium phosphate 9 g/kg, multivitamin 4 g/kg, salt 10 g/kg, multi-mineral 1 g/kg, baking soda 10 g/kg, mold inhibitor 1.5 g/kg.

#### 2.3.3 Gas collection and composition analysis

At 2, 4, 8, 12, 24, and 48 h of the experiment, the total gas production (TGP) by fermentation in each bottle was collected using a 30 mL syringe. The gas sample was stored in aluminum foil airbags until analysis. The gas composition (CH₄, CO₂, and H₂) was analyzed using an SP-2060 T Gas Chromatograph (Tianpu, China) equipped with a thermal conductivity detector (TCD), and two serially connected stainless steel columns: a 5A molecular sieve column (3 mm × 3 m, 60–80 mesh Chromosorb) followed by a TDX-01 carbon molecular sieve column (3 mm × 1 m, 60–80 mesh Chromosorb). The analysis was performed under isothermal conditions at 100°C with the detector maintained at 100°C. High-purity argon (≥99.999%) was used as carrier gas at a flow rate of 30 mL/min and operating pressure of 0.5 MPa. Gas samples (1 mL) were injected using gas-tight syringes. Quantification was achieved by external calibration with certified standard gas mixtures containing 10% CH_4_, 20% CO_2_, and 2% H_2_ in argon balance. TGP, CH_4_, CO_2_, and H_2_ volume normalized on a dry matter basis (mL/g DM).

#### 2.3.4 Fermentation substrate chemical analysis

The DM content of the seaweed and fermentation substrate was determined by achieving constant weight at 105°C (AOAC, 2000). Organic matter was measured as the loss in the muffle furnace (Yiheng, China) at 550°C for 8 h (AOAC, 2000). Neutral detergent fiber, and acid detergent fiber (NDF and ADF), were measured according to the method described by Van Soest et al. (1991) using the ANKOM 200i Fiber Analyzer (ANKOM Technology, United States). Crude protein (CP) was determined by KjelMaster K-375 (BUCHI, CH) and the nitrogen concentration was multiplied by the conversion factor of 6.25 (Mariotti et al., 2008). After the fermentation is completed, the nylon bags were rinsed with laboratory tap water until the water ran clear, then dried at 65°C for 48 h and the remaining substrate was weighed. The dry matter disappearance (DMD), neutral detergent fiber degradation (NDFD), acid detergent fiber degradation (ADFD) and crude protein degradation (CPD) during the fermentation process are calculated. The DM, NDF, ADF and CP contents in the fermentation residue were measured by the above methods.

DMD (%) was determined by weight difference before and after fermentation, with corrections made for blank bag losses.

$$\mathrm{NDFD}/\mathrm{ADFD}/CPD\;\left(\%\right)=\left[\begin{array}{l} \left(\begin{array}{l} \mathrm{initial nutrient content}-\\ \mathrm{residual nutrient content} \end{array}\right)/\\ \mathrm{initial nutrient content} \end{array}\right]\times 100.$$

#### 2.3.5 Rumen fermentation parameters analysis

After fermentation, put the bottle in an ice water bath for 30 min to stop fermentation. The pH of rumen fluid was immediately measured using a calibrated PHSJ-4F pH meter (Leici, China). The remaining fermentation substrate was collected in nylon bags for further analysis. Individual fermentation fluid samples were stored at −80°C for VFA, ammonia (NH_3_-N), rumen microbial protein (MCP), metagenomic, and metabolomics analysis. For VFA concentration analysis, the sample was centrifuged at 5,400 rpm for 10 min. Then, 1 mL of the supernatant and 0.2 mL of the metaphosphoric acid solution containing 2-ethylbutyric acid (2 g/L) as internal standard were taken, mixed, and placed in an ice water bath for more than 30 min. The VFA concentration was centrifuged again at 10,000 rpm for 10 min, and the supernatant was used to measure the VFA concentration using Agilent 6890 N (Agilent, United States). Gas chromatography operating conditions have been described by Erwin et al. (1961). NH_3_-N concentration was determined using the UV-2600 Spectrophotometer (Unico, China) method as described by Broderick and Kang (1980). MCP concentration was determined using the Coomassie Brilliant Blue method with bovine serum albumin (BSA) as standard (Sedmak and Grossberg, 1977). A standard curve (0–1,000 μg/mL BSA) was established for quantification. 100 mg Coomassie Brilliant Blue in 50 mL 95% ethanol, then adding 100 mL 85% phosphoric acid, and diluting to 1 L with distilled water. Rumen fluid samples were centrifuged at 12,000 × g for 15 min. Then, 100 μL supernatant was mixed with 1 mL dye reagent, incubated for 10 min, and measured at 595 nm Spectronic 200 (Thermo Scientific, United States).

#### 2.3.6 DNA extraction, library construction, and metagenomic sequencing

According to the manufacturer’s instructions, total genomic DNA was extracted from rumen fermentation fluid samples using the MagAtrract PowerSoil Pro DNA Kit (Omega Bio-tek, Norcross, GA, United States). DNA purity was verified with A260/A280 ratios of 1.8–2.0 and A260/A230 ratios >1.7 to exclude protein or carbohydrate contamination, coupled with TBS-380 fluorometer quantification (≥20 ng/μL).

DNA extract was fragmented to an average size of about 400 bp using Covaris M220 (Gene Company Limited, China) for paired-end library construction. Paired-end library was constructed using NEXTFLEX Rapid DNA-Seq (Bioo Scientific, Austin, TX, United States). Adapters containing the full complement of sequencing primer hybridization sites were ligated to the blunt end of fragments. Paired-end sequencing was performed on Illumina NovaSeq (Illumina Inc., San Diego, CA, United States) at Majorbio Bio-Pharm Technology Co., Ltd. (Shanghai, China) using NovaSeq 6000 S4 Reagent Kit v1.5 (300 cycles) according to the manufacturer’s instructions.^1^ Sequence data associated with this project have been deposited in the NCBI Short Read Archive database (Accession Number: PRJNA1148834).

#### 2.3.7 Quality control sample and UHPLC-MS/MS analysis

The LC-MS/MS analysis of the sample was conducted on a Thermo UHPLC-Q Exactive HF-X system equipped with an ACQUITY HSS T3 column (100 mm × 2.1 mm i.d., 1.8 μm; Waters, United States) at Majorbio Bio-Pharm Technology Co. Ltd. (Shanghai, China). Each sample was spiked with internal standards (2-chloro-L-phenylalanine, lidocaine, and glyceryl trioleate at 1 μg/mL) for quantification and system monitoring.

The pretreatment of LC/MS raw data was performed by Progenesis QI (Waters Corporation, Milford, United States) software. To control batch effects, samples were randomized across acquisition sequences with pooled quality control (QC) samples analyzed every 10 injections. Data normalization combined internal standard correction and QC-based LOESS regression. A three-dimensional data matrix in CSV format was exported. This three-dimensional matrix included: sample information, metabolite name, and mass spectral response intensity. Internal standard peaks and any known false positive peaks (including noise, column bleed, and derivatized reagent peaks) were removed from the data matrix, deredundant, and peak pooled. At the same time, the metabolites were identified by searching databases, and the main databases were the HMDB,^2^ Metlin,^3^ and Majorbio Database.

### 2.4 Statistical analysis

The resulting model was analyzed with an ANOVA. For nutrient degradation (DMD, NDFD, and ADFD), total gas produced, gas composition (CH_4_, CO_2_, and H_2_), and fermenter parameters (pH, VFA, NH_3_-N, and MCP), data were tested for normality (Shapiro–Wilk) and statistically significant mean differences were tested for using parametric ANOVA (with Dunn) as appropriate. All ANOVA analyses were performed using IBM SPSS (27.0) software. Statistically significant means were considered when *p* < 0.05, while *p* < 0.1 was considered a tendency toward statistical significance. The metagenome and metabolome data were processed by Shanghai Meiji Company. The data were analyzed on the free online platform of Majorbio Cloud Platform (https://cloud.majorbio.com, accessed May 10, 2024).

## 3 Results

### 3.1 Effect of different treatments on the bioactive components of *Asparagopsis taxiformis*

The top 10 bioactive components of the compositional ratio of *A. taxiformis* after different treatments are listed in Table 2. The main bioactive components in freeze-dried *A. taxiformis* are Bromoacetic acid (10.28%), Catechi (6.25%), Rosmarinic acid (3.48%), Dibromoiodomethane (2.91%), Coumaric acid (2.71%), P-Bromophenol (2.36%), Hesperidine (2.32%), 2,4,6-Tribromothiophenol (2.28%), Daidzein (2.26%), 2,4-Dibromophenol (1.78%), respectively.

**Table 2:** Effect of different treatments on composition of the main bioactive components (top 10) in *Asparagopsis taxiformis.*

Parameter	Percentage	CAS
Dried		
3,7-Dihydroxyflavone %	10.82	492-00-2
Gallic acid %	5.92	149-91-7
Vanillin %	5.89	121-33-5
4-Hydroxycinnamic acid %	3.91	4501-31-9
Rosmarinic acid %	3.07	20283-92-5
Salicylic acid %	2.48	69-72-7
Catechin %	2.11	18829-70-4
Kaempferol-3-glucoside %	1.69	480-10-4
Morin hydrate %	1.15	480-16-0
Epicatechin %	1.08	35323-91-2
Freeze-dried		
Bromoacetic acid %	10.28	79-08-3
Catechin %	6.25	18829-70-4
Rosmarinic acid %	3.48	20283-92-5
Dibromoiodomethane %	2.91	593-94-2
Coumaric acid %	2.71	500-05-0
P-Bromophenol %	2.36	106-41-2
Hesperidine %	2.32	520-26-3
2,4,6-Tribromothiophenol %	2.28	57730-98-0
Daidzein %	2.26	486-66-8

The main bioactive components in dried *A. taxiformis* are 3,7-Dihydroxyflavon (10.82%), Gallic acid (5.92%), Vanillin (5.89%), 4-Hydroxycinnamic acid (3.91%), Rosmarinic acid (3.07%), Salicylic acid (2.48%), Catechin (2.11%), Kaempferol-3-glucoside (1.69%), Morin hydrate (1.15%), Epicatechin (1.08%), respectively.

### 3.2 Effect of *Asparagopsis taxiformis* supplementation on gas production parameters and CH_4_ production

**Figure 1:**
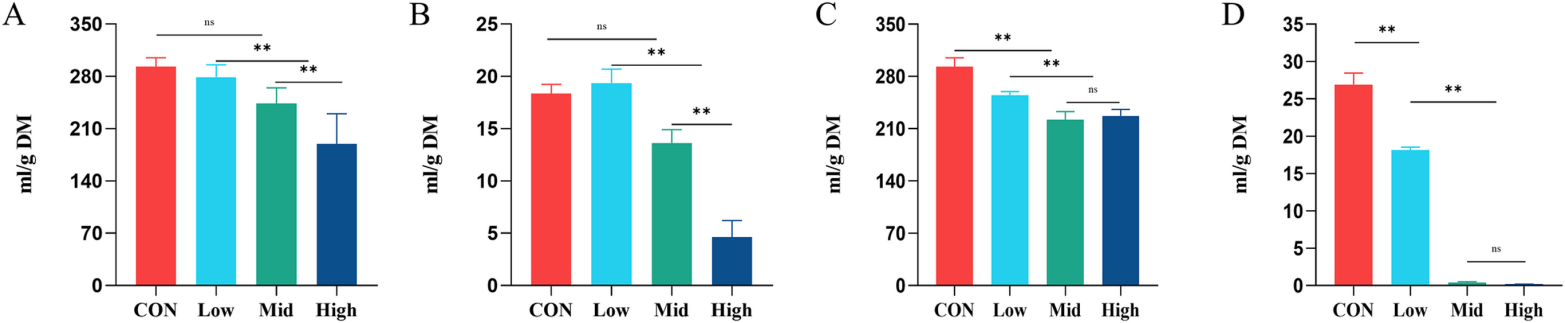
Effect of different treatments and supplementation levels of *Asparagopsis taxiformis* on total gas production (TGP) and methane (CH_4_) emission from *in vitro* rumen fermentation. **(A)** Total gas TGP at dried treatment. **(B)** CH_4_ emission at dried treatment. **(C)** TGP at freeze-dried treatment. **(D)** CH_4_ emission at freeze-dried treatment. CON, control group; Low, CON plus 2% *A. taxiformis*; Mid, CON plus 5% *A. taxiformis*; High, CON plus 10% *A. taxiformis*. ^*^<0.05, ^**^<0.01, ^***^<0.001.

In the current study, the total gas production (TGP) in the CON was 293.02 ± 12.02 mL/g DM. TGP in the Mid and High groups after supplementation dried treatment of *A. taxiformis* was 243.63 ± 21.02 and 189.72 ± 40.10 mL/g DM respectively, significantly lower than that in the CON (*p* < 0.01) (Figure 1A). Supplementation with dried *A. taxiformis* significantly reduced CH_4_ production (Figure 1B). Compared with the CH_4_ production of 26.89 ± 1.56 mL/g DM in the control group, the Low group (27.65 ± 3.21 mL/g DM) did not alleviate the methane production. The Mid group produced 17.51 ± 2.77 mL/g DM of CH_4_, and the High group produced 4.61 ± 1.07 mL/g DM. Compared with the CON, the CH_4_ production decreased by 34.52% (*p* < 0.01) and 82.89% (*p* < 0.01) in the Mid and High groups, respectively.

Unlike the dried *A. taxiformis*, the freeze-dried *A. taxiformis* showed the greater potential to reduce the TGP and CH_4_. As shown in Figure 1C, all three freeze-dried *A. taxiformis* supplementation levels significantly reduced TGP from *in vitro* rumen fermentation. Compared with CON, the TGP of Low, Mid, and High groups were decreased by 13.02% (*p* < 0.01), 24.18% (*p* < 0.01), and 22.56% (*p* < 0.01), respectively. The Low group produced 18.11 ± 0.45 mL/g DM of CH_4_, and the reduction effect reached 32.44% (*p* < 0.01). The CH_4_ production was almost completely suppressed in the Mid and High groups, and only 0.38 ± 0.12 and 0.17 ± 0.02 mL/g DM CH_4_ were produced. The CH_4_ reduction reached 98.53% (*p* < 0.01) and 99.33% (*p* < 0.01), respectively (Figure 1D).

In the current study, the potential of freeze-dried *A. taxiformis* to mitigate methane emissions is significantly better than that of dried treatment at the same supplementation level. When supplementation dried *A. taxiformis*, the H_2_ production increased significantly only in the High group (*p* < 0.01). When supplementing the freeze-died treatment of *A. taxiformis*, the H_2_ production increased significantly in both the Mid group (*p* < 0.01) and High group (*p* < 0.01). Furthermore, the production of CO_2_ was significantly reduced only in the freeze-dried treatment High group (*p* < 0.01). Supplementary Table S1 presents the effect of *A. taxiformis* supplementation on the gas production parameters observed in this study.

### 3.3 Effect of *Asparagopsis taxiformis* supplementation on nutrient degradation

Supplementing with *A. taxiformis* significantly inhibits CH_4_ emissions, making it necessary to investigate further whether its supplementation negatively affects nutrient degradation and rumen fermentation and explore the mechanisms behind the reduction in CH_4_ emissions. The negative impact of *A. taxiformis* supplementation on nutrient degradation was affected by the treatment method and supplementation level. As shown in Table 3, when the supplementation level was greater than 5%, a decrease in DMD, NDFD, ADFD, and CPD was observed in both dried and freeze-dried treatments. A high level of supplementation significantly inhibits the degradation of these nutrients. In the dried treatment, the High group reduced DMD, NDFD, ADFD, and CPD by 3.69% (*p* < 0.01), 11.25% (*p* < 0.01), 7.70% (*p* < 0.01), and 36% (*p* < 0.01), respectively.

**Table 3:** Effect of the two treatments and different supplementation levels *Asparagopsis taxiformis* on nutrient degradation *in vitro* rumen fermentation.

Parameter	CON	Low	Mid	High	*p*
Dried					
DMD %	86.45 ± 1.01^AB^	87.54 ± 0.82^A^	85.53 ± 1.19^B^	83.26 ± 1.84^C^	<0.001
NDFD %	69.21 ± 2.31^A^	65.97 ± 2.25^AB^	63.04 ± 3.05^BC^	61.42 ± 4.25^C^	0.002
ADFD %	65.50 ± 2.59^A^	64.13 ± 2.37^AB^	51.34 ± 4.01^C^	60.45 ± 4.36^BC^	<0.001
CPD %	81.83 ± 1.36^A^	81.61 ± 1.21^A^	77.46 ± 1.86^B^	52.37 ± 5.25^C^	<0.001
Freeze-dried					
DMD %	86.45 ± 1.01^ab^	87.04 ± 0.93^a^	84.93 ± 1.95^b^	84.61 ± 1.28^b^	0.015
NDFD %	69.21 ± 2.31	69.99 ± 2.16	66.16 ± 4.38	65.84 ± 2.86	0.06
ADFD %	65.50 ± 2.59^ab^	68.00 ± 2.30^a^	61.53 ± 4.98^b^	62.36 ± 3.15^b^	0.014
CPD %	81.83 ± 1.36^A^	82.47 ± 1.26^A^	51.26 ± 6.31^B^	48.45 ± 4.32^B^	<0.001

DMD, degradation of dry matter; NDFD degradation of neutral detergent fiber; ADFD, degradation of acid detergent fiber; CPD, degradation of crude protein; CON, control group; Low, CON plus 2% *A. taxiformis*; Mid, CON plus 5% *A. taxiformis*; High, CON plus 10% *A. taxiformis*. ^ab^Means bearing different superscripts in the same row differ significantly (*p* < 0.05). ^A,B^Means bearing different superscripts in the same row differ significantly (*p* < 0.01).

In addition, with supplementation freeze-dried *A. taxiformis*, the inhibitory effect on degradation of nutrients except CP is alleviated, and the high group reduced DMD, NDFD, and ADFD by 2.12% (*p* < 0.01), 4.86% (*p* < 0.01), and 4.79% (*p* < 0.01), respectively. It is worth noting that CPD was significantly reduced in both freeze-dried Mid and High groups, which were reduced by 37.35% (*p* < 0.01) and 40.79% (*p* < 0.01) compared with the CON.

### 3.4 Effect of *Asparagopsis taxiformis* supplementation on rumen fermentation parameters

Rumen fermentation parameters are important indicators of rumen internal environment stability and can reflect changes in rumen fermentation function. Adding *A. taxiformis* affects rumen CH_4_ production and rumen fermentation parameters from *in vitro* rumen fermentation (Table 4). After the dried treatment, the supplementation of *A. taxiformis* in the Low and Mid groups significantly reduced the pH of rumen fluid (*p* < 0.01). NH_3_-N, MCP, and total VFA concentrations were not affected, but individual VFA concentrations changed along with supplementation levels. The acetate, isobutyrate, and isovalerate concentrations were all significantly reduced (*p* < 0.05). The Mid group produced 32.73 ± 3.87 mmol/L of acetate and 0.83 ± 0.11 mmol/L of isobutyrate, and the High group produced 1.35 ± 0.14 mmol/L of isovalerate both of which were the lowest levels in the dried. The decrease in acetate concentration was the main reason for the lowest total VFA in the Mid group. The ratio of acetate to propionate (A:P ratio) in the CON was 2.65, and with increasing supplementation levels, the A:P ratio decreased to 2.26, 2.19, and 1.99 (*p* = 0.04), respectively.

**Table 4:** Effect of the two treatments and different supplementation levels *Asparagopsis taxiformis* on pH, NH3-N, MCP, and VFA profiles *in vitro.*

Parameter	CON	Low	Mid	High	*p*
Dried					
pH	6.76 ± 0.04^A^	6.60 ± 0.03^C^	6.66 ± 0.02^B^	6.72 ± 0.04^A^	<0.001
NH_3_-N mmol/L	18.58 ± 1.25	19.11 ± 4.30	18.27 ± 6.70	13.85 ± 4.44	0.206
MCP μg/mL	101.36 ± 7.33	100.2 ± 19.27	115.2 ± 27.36	122.2 ± 14.57	0.147
Total VFA mmol/L	72.57 ± 7.82	65.80 ± 10.01	57.72 ± 6.79	67.12 ± 4.00	0.069
Acetate mmol/L	43.73 ± 5.04^a^	38.99 ± 6.27^ab^	32.73 ± 3.87^b^	36.67 ± 1.95^ab^	0.02
Propionate mmol/L	16.45 ± 1.53	15.55 ± 2.09	14.89 ± 1.75	18.39 ± 1.32	0.052
Isobutyrate mmol/L	1.08 ± 0.10^a^	0.96 ± 0.15^ab^	0.83 ± 0.11^b^	0.87 ± 0.72^b^	0.028
Butyrate mmol/L	8.09 ± 0.83	7.37 ± 1.24	6.67 ± 0.82	8.55 ± 0.72	0.056
Isovalerate mmol/L	1.85 ± 0.19^A^	1.65 ± 0.29^AB^	1.39 ± 0.18^B^	1.35 ± 0.14^B^	0.009
Valerate mmol/L	1.35 ± 0.11	1.25 ± 0.17	1.17 ± 0.13	1.27 ± 0.10	0.296
A:P ratio	2.65 ± 0.06^a^	2.26 ± 0.58^ab^	2.19 ± 0.08^ab^	1.99 ± 0.10^b^	0.042
Freeze-dried					
pH	6.76 ± 0.04^a^	6.76 ± 0.02^a^	6.69 ± 0.01^b^	6.71 ± 0.05^ab^	0.015
NH_3_-N mmol/L	18.58 ± 1.25^A^	17.16 ± 3.08^A^	12.11 ± 1.04^B^	13.72 ± 2.62^B^	<0.001
MCP μg/mL	101.36 ± 7.33^b^	121.20 ± 25.04^ab^	135.86 ± 19.38^a^	109.20 ± 12.21^b^	0.014
Total VFA mmol/L	72.57 ± 7.82	68.25 ± 7.59	63.85 ± 11.68	57.64 ± 14.93	0.141
Acetate mmol/L	43.73 ± 5.04^A^	39.15 ± 4.65^AB^	33.82 ± 6.29^BC^	29.51 ± 7.72^C^	0.003
Propionate mmol/L	16.45 ± 1.53	17.47 ± 1.77	17.45 ± 3.09	16.15 ± 3.79	0.768
Isobutyrate mmol/L	1.08 ± 0.10^A^	0.97 ± 0.94^A^	0.77 ± 0.12^B^	0.76 ± 0.18^B^	0.001
Butyrate mmol/L	8.09 ± 0.83	7.64 ± 0.82	8.65 ± 1.78	8.78 ± 2.87	0.668
Isovalerate mmol/L	1.85 ± 0.19^A^	1.68 ± 0.17^A^	2.00 ± 0.34^A^	1.30 ± 0.29^B^	0.001
Valerate mmol/L	1.35 ± 0.11	1.32 ± 0.11	1.14 ± 0.16	1.12 ± 0.24	0.061
A:P ratio	2.65 ± 0.06^A^	2.23 ± 0.05^B^	1.93 ± 0.06^C^	1.81 ± 0.05^D^	<0.001

A:P ratio, Acetate/Propionate ratio; CON, control group; Low, CON plus 2% *A. taxiformis*; Mid, CON plus 5% *A. taxiformis*; High, CON plus 10% *A. taxiformis*. ^ab^Means bearing different superscripts in the same row differ significantly (*p* < 0.05). ^AB^Means bearing different superscripts in the same row differ significantly (*p* < 0.01).

The changes in pH and NH_3_-N in the freeze-dried maintained the same trend, both decreasing in the Mid and High groups and lowest in the Mid group (Table 4). The NH_3_-N concentration in the Mid and High groups was reduced by 34.82% (*p* < 0.01) and 26.15% (*p* < 0.01) compared with the CON. However, the MCP concentration in the Mid group was 135.86 ± 19.38 μg/mL, which was 34.03% (*p* < 0.05) higher than that in the CON (101.36 μg/mL). The concentration of total VFA has a downward trend with increasing supplementation levels, but it is not significant (*p* = 0.141). Increased supplementation levels caused significant decreases in acetate (*p* < 0.01), isobutyrate (*p* < 0.01), and isovalerate (*p* < 0.01) concentrations. In the High group, the concentration of acetate was 29.51 ± 7.72 mmol/L, which was 32.51% lower than the 43.73 ± 5.04 mmol/L in the CON, while isobutyrate and isovalerate also decreased by 29.62% and 29.72%. In the freeze-dried treatment, the acetate is significantly reduced, but the propionate does not change significantly, which causes a significant decrease in the A:P ratio (*p* < 0.01). After supplementing three levels of *A. taxiformis*, the A:P ratios were 2.23, 1.93, and 1.81, respectively.

Based on the above experimental results, we suggest that freeze-dried treatment of *A. taxiformis* is more effective in alleviating CH_4_ emissions. However, the High group showed a huge negative impact on rumen fermentation and was not fit for application, thus we selected the Low and Mid groups samples for metagenomic testing to further analyze the function and mechanism of *A. taxiformis* in decreasing CH_4_ production.

### 3.5 Effect of *Asparagopsis taxiformis* supplementation on the composition of microbial communities

#### 3.5.1 Bacterial community effects under *Asparagopsis taxiformis* supplementation

**Figure 2:**
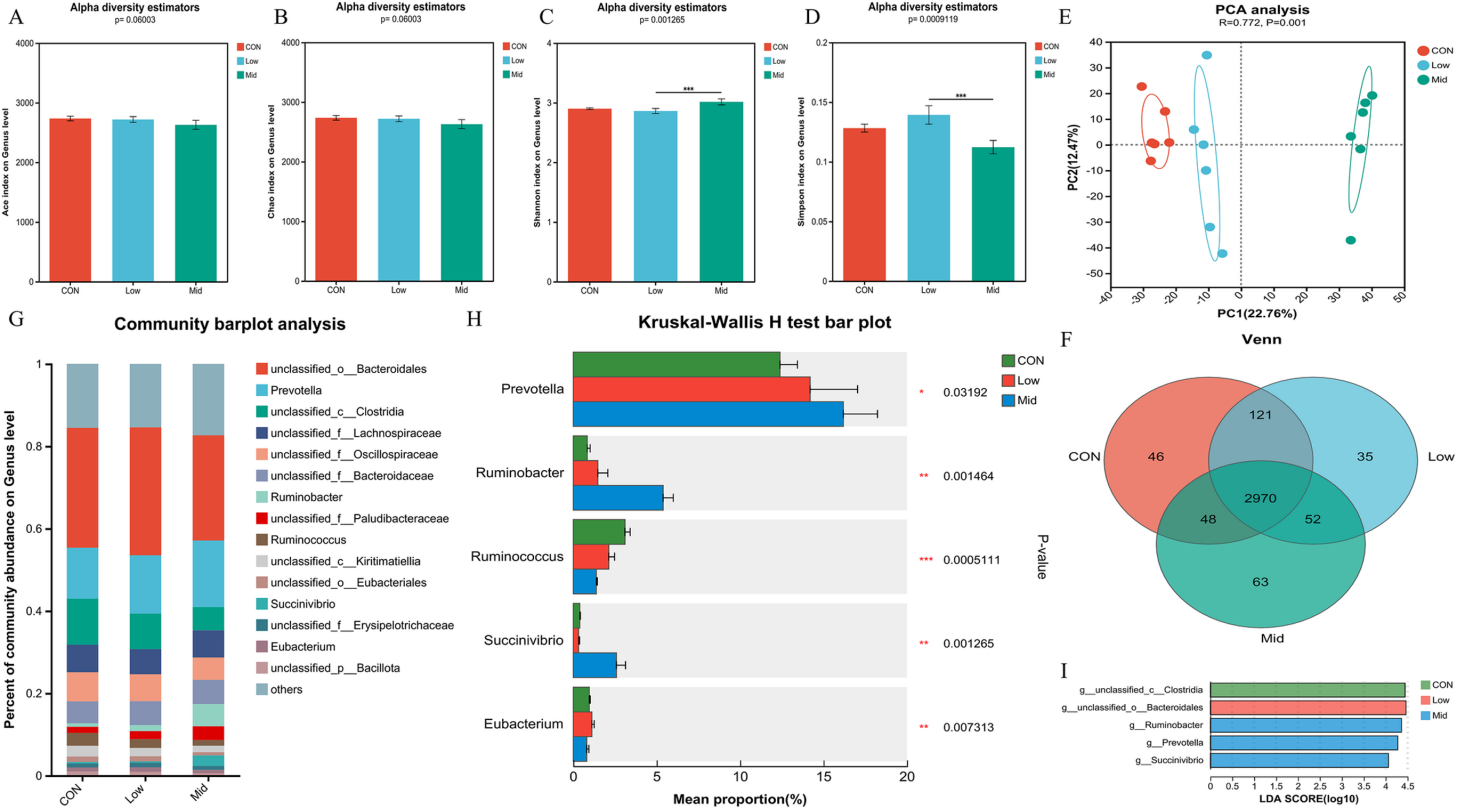
Effect of different treatments and supplementation levels of *Asparagopsis taxiformis* on rumen bacterial composition in the *in vitro* rumen fermentation. **(A)** Ace index on genus level. **(B)** Chao index on genus level. **(C)** Shannon index on genus level. **(D)** Simpson index on genus level. **(E)** Beta diversity. **(F)** Venn diagram on genus level. **(G)** Relative abundances of the 15 most abundant genus-level across all three groups. **(H)** Differences in bacterial genus levels by metagenomics sequencing. **(I)** The LDA values of different species among the three groups on genus level (LDA > 4). CON, control group; Low, CON plus 2% *A. taxiformis*; Mid, CON plus 5% *A. taxiformis*; High, CON plus 10% *A. taxiformis*. ^*^<0.05, ^**^<0.01, ^***^<0.001.

The study mainly observed the effect of supplementing *A. taxiformis* on the community structure of rumen bacteria at the genus level. In terms of bacterial *α*-diversity, the Ace index (Figure 2A) and Chao index (Figure 2B) had no significant differences among the three groups (*p* > 0.05). The Mid group significantly increased the Shannon index (*p* < 0.01) (Figure 2C). It also significantly decreased the Simpson index (*p* < 0.01) (Figure 2D), but there was no significant difference between the CON and Low groups. In terms of bacterial *β*-diversity, the results showed that the microorganisms in the three groups were separated (R = 0.772, *p* < 0.01) (Figure 2E). The Venn diagram shows that at the genus level, the three groups have a total of 2,970 common species. There were 46, 35, and 63 unique species in the CON, Low, and Mid groups, respectively (Figure 2F). At the genus level, we analyzed the ruminal top 15 species in relative abundance. Among the annotated species, *Prevotella*, *Ruminobacter*, *Ruminococcus*, *Succinivbrio*, and *Eubacterium* are the dominant genera in the three groups (Figure 2G). Further observation of the effect of *A. taxiformis* supplementation on the microbial bacterial community structure revealed that the relative abundance of the dominant bacterial genera was significantly influenced by the level of freeze-dried *A. taxiformis* supplementation (Figure 2H). Compared with the CON group, the Mid group significantly increased the relative abundance of *Prevotella* (*p* < 0.05). Compared with the other two groups, the Mid group had a significantly higher relative abundance of *Ruminobacter* (*p* < 0.01), and *Succinivbrio* (*p* < 0.01), and there was no difference between the CON and Low groups (*p* > 0.05). The relative abundance of *Ruminococcus* significantly decreased along with increasing levels of *A. taxiformis* supplementation in the three groups (*p* < 0.01). The relative abundance of *Eubacterium* in the Mid group was not different from that in the CON, but was significantly lower than in the Low group (*p* < 0.01). When the LDA threshold is >4, we found that *Prevotella*, *Ruminobacter*, and *Succinivbrio* were significantly enriched, indicating that their relative abundance increased significantly in the Mid group (Figure 2I). The changes in bacterial communities at the phylum and species levels are shown in Supplementary Figures S1, S2.

#### 3.5.2 Archaeal community effects under *Asparagopsis taxiformis* supplementation

**Figure 3:**
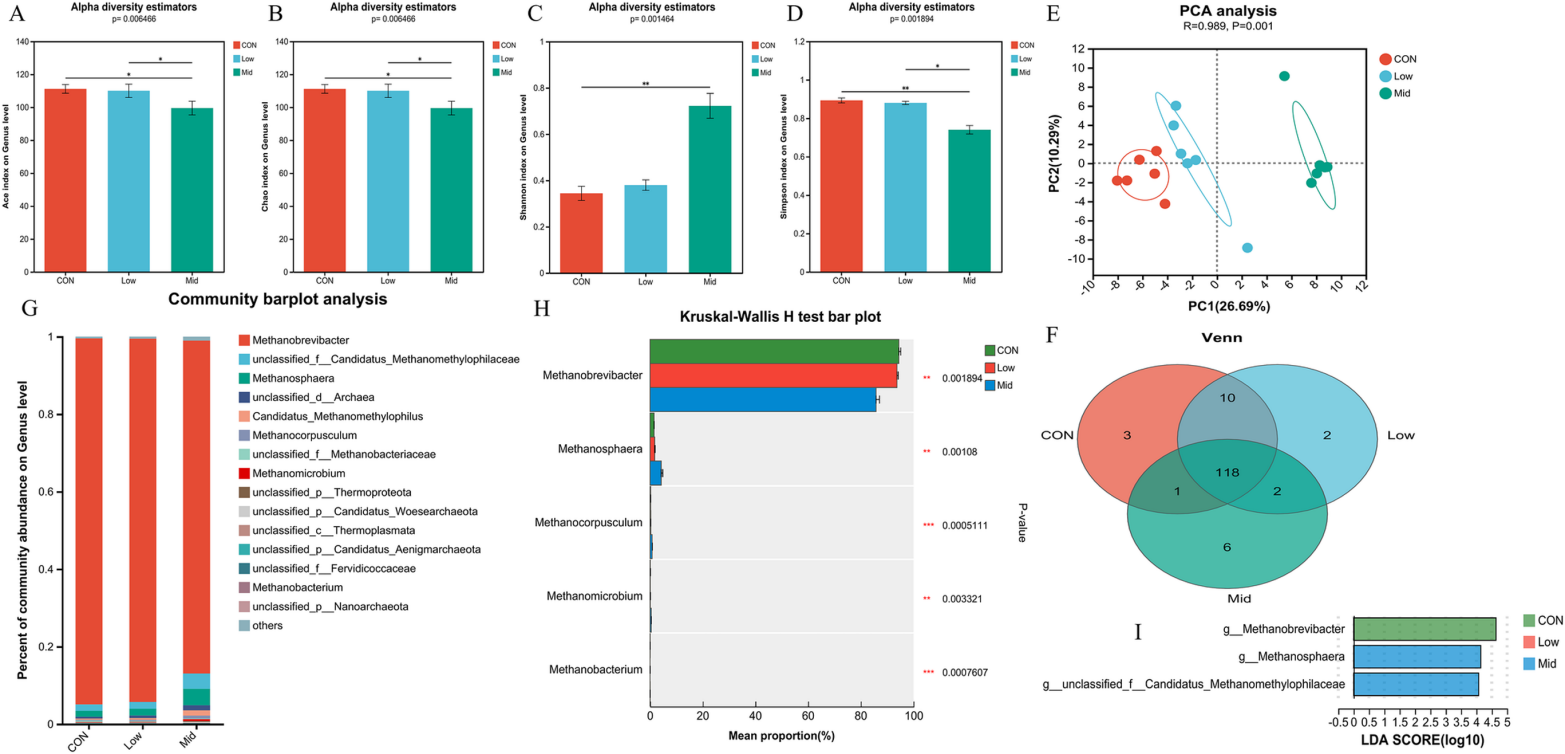
Effect of different treatments and supplementation levels of *Asparagopsis taxiformis* on rumen archaea composition in the *in vitro* rumen fermentation. **(A)** Ace index on genus level. **(B)** Chao index on genus level. **(C)** Shannon index on genus level. **(D)** Simpson index on genus level. **(E)** Beta diversity. **(F)** Venn diagram on genus level. **(G)** Relative abundances of the 15 most abundant genus-level across all three groups. **(H)** Differences in archaea genus levels by metagenomics sequencing. **(I)** The LDA values of different species among the three groups on genus level (LDA > 4). CON, control group; Low, CON plus 2% *A. taxiformis*; Mid, CON plus 5% *A. taxiformis*; High, CON plus 10% *A. taxiformis*. ^*^<0.05, ^**^<0.01, ^***^<0.001.

The effect of *A. taxiformis* supplementation on the archaeal community were also observed at the genus level. Different from bacteria, Ace (Figure 3A), Chao (Figure 3B), and Simpson (Figure 3D) indexes were all significantly decreased (*p* < 0.01), and the Shannon (Figure 3C) index was significantly increased (*p* < 0.01) in the Mid group. However, there was no difference between the Low and CON groups. The result of PCA principal component analysis showed that the archaeal communities of the three groups of samples were separated (R = 0.989, *p* < 0.01) (Figure 3E). The result of the Venn diagram showed that there were 118 archaeal species in the three groups (Figure 3F), with 3, 2, and 6 unique archaeal species, respectively. *Methanobrevibacter* and *Methanosphaera* are the dominant genera at the genus level (Figure 3G). The species difference histograms (Figure 3H) illustrate that there is no significant difference in the relative abundance of archaea in the CON and Low groups. However, in the Mid group, the relative abundance of *Methanobrevibacter* (*p* < 0.01) was significantly decreased, and the abundance of *Methanosphaera* (*p* < 0.01), *Methanocorpusculum* (*p* < 0.01), and *Methanomicrobium* (*p* < 0.01) were significantly increased. Setting the LDA score >4, the results also showed that *Methanobrevibacter* was significantly enriched in the CON group, and *Methanosphaera* was significantly enriched in the Mid group, which is consistent with the species difference results (Figure 3I). Supplementary Figures S3, S4 show the changing trends in archaea at the phylum and species level.

### 3.6 Effect of *Asparagopsis taxiformis* supplementation on KEGG pathways in rumen

#### 3.6.1 KEGG functional pathway enrichment analysis

**Figure 4:**
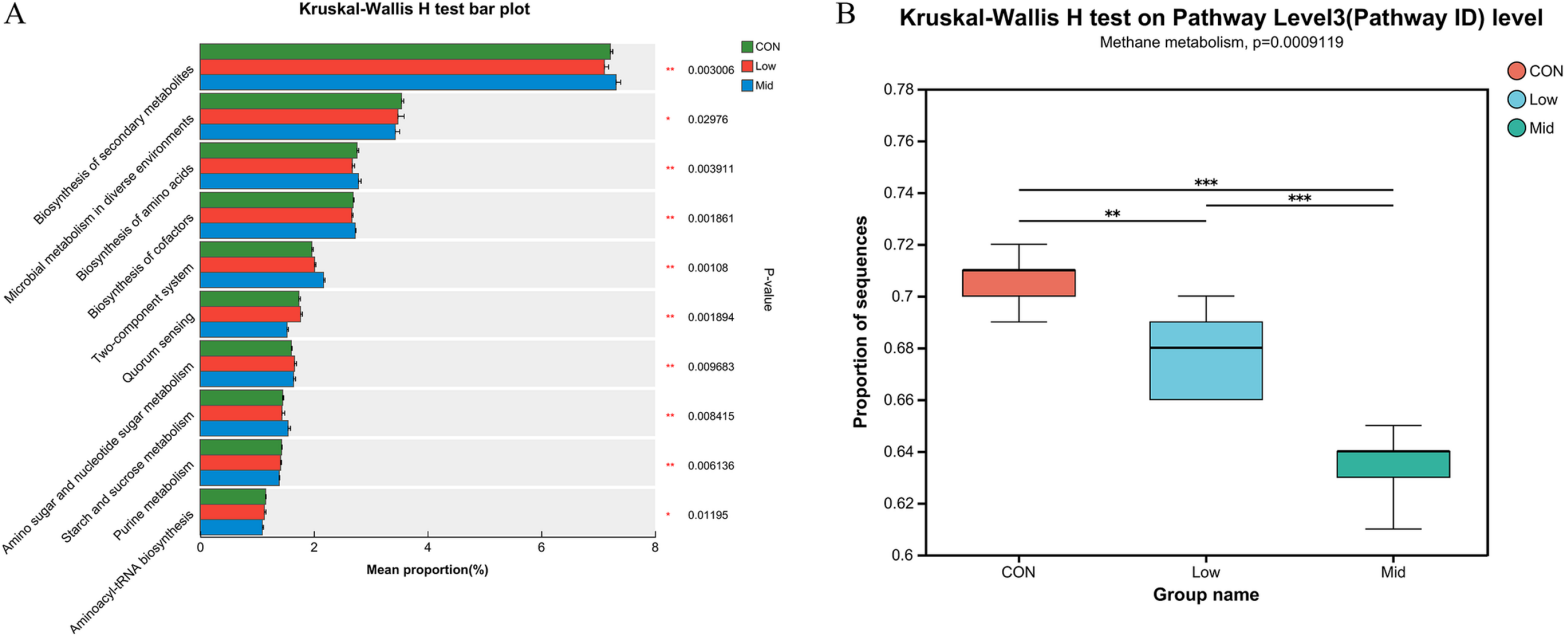
Effect of different levels of freeze-dried treatment *Asparagopsis taxiformis* in the *in vitro* rumen fermentation KEGG pathway. **(A)** Differences in pathway level 3 (Pathway ID). **(B)** Differences in methane metabolism among the three groups. CON, control group; Low, CON plus 2% *A. taxiformis*; Mid, CON plus 5% *A. taxiformis*; High, CON plus 10% *A. taxiformis*. ^*^<0.05, ^**^<0.01, ^***^<0.001.

**Figure 5:**
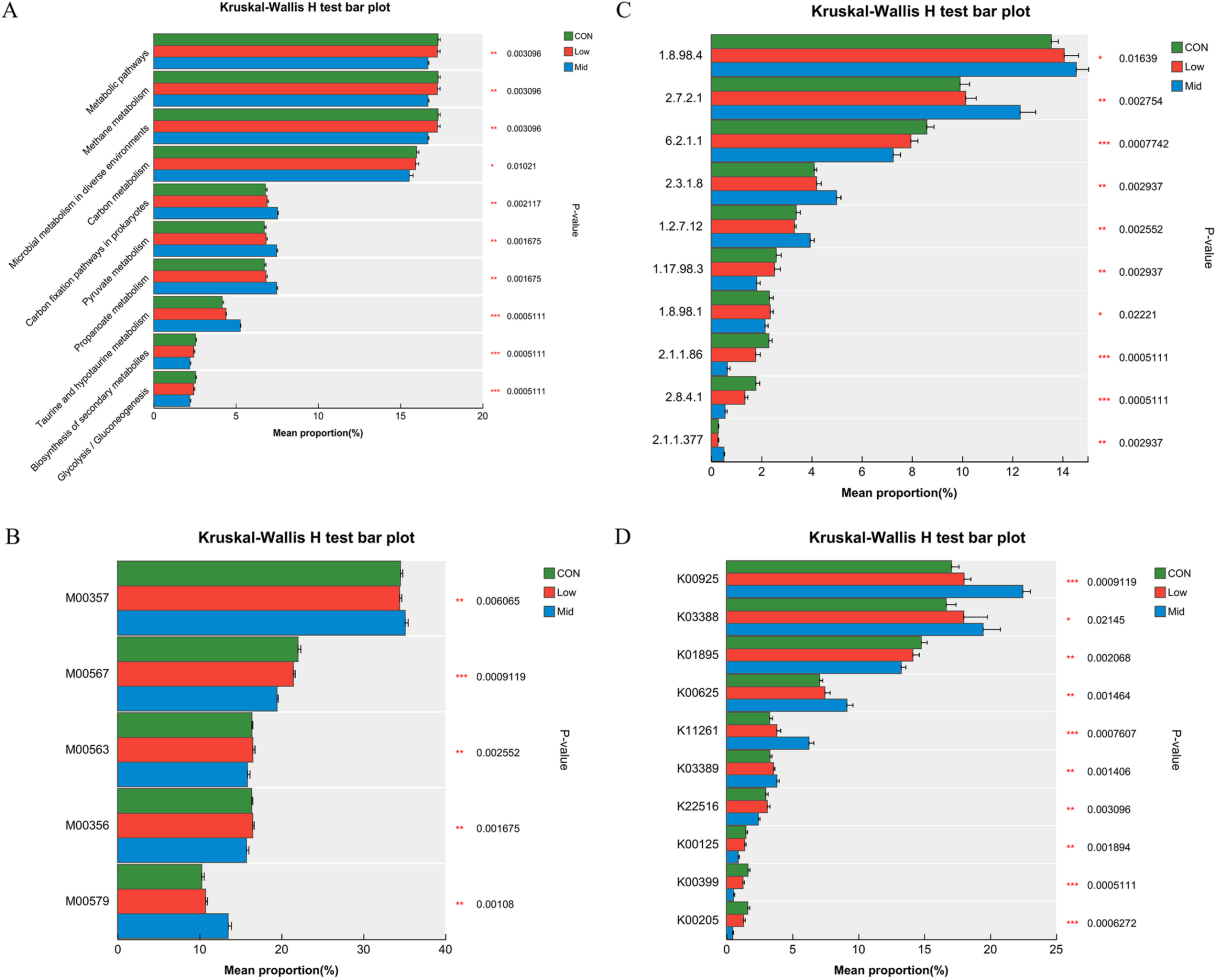
Effect of different levels of *Asparagopsis taxiformis* supplementation on methane metabolism function level. **(A)** Differences in methane metabolism at Pathway level 3. **(B)** Differences in methane metabolism at Module. **(C)** Differences in methane metabolism at Enzyme. **(D)** Differences in methane metabolism at KO (KEGG Orthology). CON, control group; Low, CON plus 2% *A. taxiformis*; Mid, CON plus 5% *A. taxiformis*; High, CON plus 10% *A. taxiformis*. ^*^<0.05, ^**^<0.01, ^***^<0.001.

To further investigate the reasons for the reduction in methane emissions, the effect of *A. taxiformis* supplementation on carbon metabolism and methane metabolism in different groups were analyzed. As shown in Figure 4A, the relative abundance of some KEGG pathways such as the Biosynthesis of secondary metabolites, Biosynthesis of amino acids, and Biosynthesis of cofactors increased significantly (*p* < 0.01). In contrast, Microbial metabolism in diverse environments, Quorum sensing, and Purine metabolism decreased significantly (*p* < 0.05) in the Mid group. We primary focus on the changes in the CH_4_ metabolism pathway, the relative abundance of methane metabolism significantly decreased with increasing supplementation levels of *A. taxiformis* (*p* < 0.01) (Figure 4B). Gene sets related to methane metabolism were screened for further analysis. In the Mid group, the relative abundance of Metabolic pathways, Methane metabolism, Microbial metabolism in diverse environments, Carbon metabolism, Biosynthesis of secondary metabolites, and Glycolysis/Gluconeogenesis was significantly reduced (*p* < 0.01), and Carbon fixation pathways in prokaryotes, Pyruvate metabolism, Propanoate metabolism, and Taurine and hypotaurine metabolism was significantly increased (*p* < 0.01) (Figure 5A).

#### 3.6.2 Enrichment analysis of KEGG pathway related to CH_4_ metabolism

Furthermore, the reasons for reduced CH_4_ production were explored at different functional levels. The impact on CH_4_ metabolism resulted in changes at the module, enzyme, and KEGG Orthology (KO) levels. The top 10 relative abundances of each function were analyzed. M00357 and M00579 significantly increased by the Mid group of *A. taxiformis* (*p* < 0.01). However, M00567, M00563, and M00356 are significantly decreased (*p* < 0.01) (Figure 5B). Figure 5C shows the change in the relative abundance of enzymes in the pathway. The relative abundance of enzymes such as 1.8.98.4 (coenzyme F420), 2.7.2.1 (acetate kinase), and 2.3.1.8 (phosphate acetyltransferase) were significantly increased (*p* < 0.05), while those of 1.17.98.3 (formate dehydrogenase) and 1.8.98.1 (dihydromethanophenazine) were significantly decreased (*p* < 0.05). It is worth noting that 6.2.1.1 (acetyl coenzyme A synthetase), 2.1.1.86, and 2.8.4.1 (methyl-CoM reductase) were significantly reduced (*p* < 0.01) in the two groups of supplementing *A. taxiformis*, but it was more significant in the Mid group. The relative abundance of K00925 (acetate kinase), K03388 (heterodisulfide reductase subunit A2), K00625 (phosphate acetyltransferase), K11261 (formylmethanofuran dehydrogenase subunit E), and K03389 (heterodisulfide reductase subunit B2) gradually increases (p < 0.01), while the relative abundance of K01895 (acetyl-CoA synthetase), K22516 (coenzyme F420 alpha subunit), K00125 (coenzyme F420 beta subunit), K00399 (methyl-coenzyme M reductase alpha subunit), and K00205 (4Fe-4S ferredoxin) gradually decreases (*p* < 0.01) (Figure 5D).

### 3.7 Effect of *Asparagopsis taxiformis* in different supplementation levels respond to rumen metabolome

**Figure 6:**
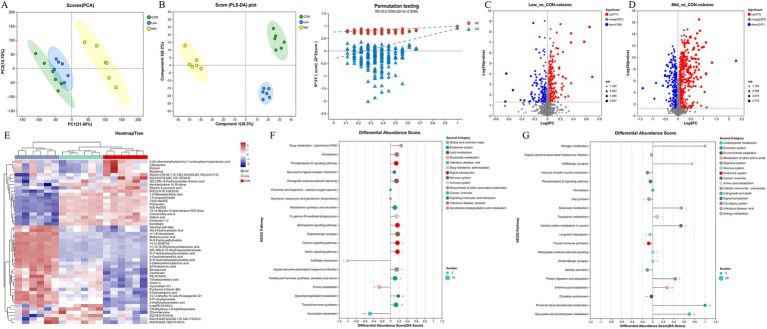
Effect of *Asparagopsis taxiformis* supplementation on the metabolomics of rumen fluid from *in vitro* rumen fermentation. **(A)** Principal component analysis (PCA) scores of metabolites in rumen fluid at different supplementation levels. **(B)** PLS-DA scores plots. R2 was 0.7556 and Q2 was 0.3098. **(C)** Volcano plot of differential metabolites between Low and CON groups. **(D)** Volcano plot of differential metabolites between Mid and CON groups. **(E)** Hierarchical clustering analysis of differential metabolites in rumen fluid at different levels of *A. taxiformis* supplementation. **(F)** Diagram of enrichment scores of differential metabolites in functional pathways in the Low group compared with the CON. **(G)** Diagram of enrichment scores of differential metabolites in functional pathways in the Mid group compared with the CON. CON, control group; Low, CON plus 2% *A. taxiformis*; Mid, CON plus 5% *A. taxiformis*; High, CON plus 10% *A. taxiformis*. ^*^<0.05, ^**^<0.01, ^***^<0.001.

Further analysis was conducted to investigate the effect of supplementing different levels of *A. taxiformis* on the *in vitro* rumen fermentation metabolites. Using the anion and cation mix combined analysis method, unit variance conversion, and confidence level 0.95, PCA analysis was performed on the metabolite composition of the CON, Low, and Mid groups. The results indicate that the separation trend of the Mid group from the CON and Low groups is obvious, indicating that in the Mid group, metabolites changed significantly (Figure 6A). The PLS-DA results showed that the metabolites of the mixed mode of CON, Low, and Mid groups were completely separated, indicating that the supplementation of *A. taxiformis* significantly disrupted the *in vitro* rumen fermentation metabolite profile (Figure 6B). The PLS-DA simulation verification results showed that the R2 of the mixed mode was above Q2 (Figure 6B), the model fit was good, the predictability was strong, and it was suitable for subsequent data analysis. According to the PLS-DA results, the differential metabolites screened out by the projected variable importance (VIP) of the multivariate analysis PLS-DA model were combined with the fold change and *p* value (VIP > 1, *p* < 0.05) as the difference between the two groups. Figure 6C shows that the Low group had 311 metabolites significantly upregulated and 168 downregulated compared to the CON. In the Mid group, *A. taxiformis* supplementation led to the up-regulation of 516 metabolites and the down-regulation of 247 metabolites (Figure 6D). Detailed changes in metabolites are provided in Supplementary Tables S2, S3.

To visualize relevant differences in the *in vitro* ruminal fermentation metabolites, hierarchical cluster analysis was performed using heatmaps (Figure 6E). Five distinct clusters were formed among these differential metabolites. The results showed that 24 metabolites, including Ascorbyl palmitate, (R)-2-Hydroxystearic Acid, and Methylsuccinic Acid, could be classified into cluster 3. The expression of metabolites in cluster 3 in the Mid group was significantly higher than in the CON and Low groups. At the same time, 16 metabolites such as Corchorifatty acid A can be classified into cluster 2. The expression of metabolites in cluster 2 in the Mid group was lower than in the other two groups. The two groups were compared with CON to analyze the effect of *A. taxiformis* supplementation on the enrichment of differential metabolites in KEGG pathways. The differences in differential metabolite expression in the KEGG pathways are illustrated for the Low group (Figure 6F) and the Mid group (Figure 6G), compared to the CON group.

## 4 Discussion

The main objectives of this study were to evaluate the effect of different supplementation levels of *A. taxiformis* in different treatments on reducing rumen fermentation CH_4_ production and to explore the mechanisms behind CH_4_ emissions. It is worth noting that the freeze-dried treatment demonstrated a stronger inhibitory effect on rumen fermentation CH_4_ production at supplementation levels ≥5% (Mid and High), reducing CH_4_ production by more than 98% (Figure 1). The result agrees with the report by Roque et al. (2019) who reported a 95% reduction in CH_4_ production with 5% supplementation of organic matter from *A. taxiformis*. Although the dried treatment of *A. taxiformis* ≥5% (Mid and High), showed a lower effect than freeze-dried treatment, it still reduced CH_4_ emissions by 34.52% to 82.89%.

The high efficiency of freeze-dried *A. taxiformis* in mitigating CH_4_ was expected. It is well known that freeze-dried can inhibit mutation and protect cell structure during the sublimation process, which can retain bioactive components to the maximum extent (Ratti, 2001; Vucko et al., 2017). This preservation would help maintain the content of haloalkanes, which were key ingredient in mitigating CH_4_ in *A. taxiformis*. Dibromoiodomethane as a haloalkanes, is a structural analog of MCR and competes to inhibit methyl transfer reactions during methanogenesis (Liu et al., 2011), and P-Bromophenol and 2,4,6-Tribromothiophenol and 2,4-Dibromophenol as polyphenol derivatives compete to inhibit the transfer of the H_2_ to the pathway of CH_4_ production, which are all reactions that further strengthen the inhibitory effect on CH_4_ (Ramdani et al., 2023).

In contrast, dried reduces the concentration of heat-sensitive phytochemicals, such as haloalkanes and polyphenol, which are critical for the antimethanogenic activity. This is the main reason why relevant bioactive components were not detected in the dried treatment. The loss of these compounds occurs due to thermal instability and oxidative reactions during dehydration (Wong and Cheung, 2001; Magnusson et al., 2014; Ling et al., 2015). In addition, dried alters the composition of nutrients such as proteins and the integrity of the cell wall, which can exacerbate the volatilization of bioactive components from *A. taxiformis* and reduce their potential as CH_4_ inhibitors (Ling et al., 2015).

In this experiment, the supplementation levels of dried treatment reduced CH_4_ by 34.52%, and 82.89%, respectively, while freeze-dried treatment reduced CH_4_ by 32.44%, 98.53%, and 99.33%, respectively. These results indicate that at the same level, freeze-dried treatment has greater potential to mitigate CH_4_, consistent with the above description that freeze-dried can better preserve bioactive components. However, a limitation of this study is that the CHBr_3_ concentrations in the different treatments and supplementation levels were not tested, preventing the provision of concrete evidence regarding changes in CHBr_3_ concentration.

The degradation of dietary nutrients is an important indicator to measure the degree of nutrient utilization by animals (Li et al., 2023). The results demonstrate that both supplementation level and treatment method influence nutrient degradation. It is noteworthy that significant differences were observed in ADFD (61.53 vs. 51.34) and CPD (51.26 vs. 77.46) between the freeze-dried and dried treatments under mid-level supplementation. The freeze-dried treatment may enhance the digestibility of ADF while preserving a higher amount of rumen-protected protein. When the supplementation level of *A. taxiformis* is more than 5%, both treatment methods significantly negative affect nutrient degradation, which is consistent with previous study results (Kinley et al., 2016a). The additional level of *A. taxiformis* more than 5% may affect the fermentation stability and inhibit the activity of carbohydrate digestive enzymes, thus affecting the degradation of nutrients (Abbott et al., 2020). CH_4_ is the final product of anaerobic fermentation of carbohydrates in the rumen of ruminants, and H_2_ plays an important role in this process (Antonius et al., 2023). In the rumen, propionate production consumes H_2_ and this pathway is considered an important H_2_ sink (Ungerfeld, 2020; Wasson et al., 2022). Therefore, changes in VFA fermentation patterns will also affect CH_4_ emissions (Sena et al., 2024). In this study, we observed no significant effect on total VFA concentration, although there was a downward trend with increasing supplementation levels in the freeze-dried treatment (Table 4). This study observed that acetate concentration was lower in the freeze-dried treatment compared to the dried treatment, with acetate concentration decreasing by 22.62%–32.51% as supplementation levels increased. Propionate concentration had no effect in all treatment groups compared to the CON. In the dried treatment, only the A:P ratio in the High group significantly decreased by 24.90%. In the freeze-dried treatment, the A:P ratio decreased by 15.84%, 27.17%, and 31.69% along with increasing supplement levels. The A:P ratio directly reflects the net balance of H_2_ during rumen fermentation, indicating the trend of methanogenic substrates and thus the potential of strategies to mitigate CH_4_ emissions (Machado et al., 2016a). This study indicated that the decrease in acetate concentration after supplementation with *A. taxiformis* might be the main reason for the decrease in the A:P ratio, and this effect was more effective in the freeze-dried treatment than in the dried treatment, and also more effective in the high supplementation level than in the low supplementation level.

In the current study, NH_3_-N concentration was significantly reduced only in the freeze-dried Mid and High groups. Several factors such as CPD, protozoa number, and MCP synthesis in the rumen of ruminants jointly affect the concentration of NH_3_-N (Kelln et al., 2023). In the freeze-dried treatment, the Low and Mid groups exhibited significantly higher MCP concentrations compared to the CON group. This result may be due to the freeze-dried *A. taxiformis* affecting the rumen environment, accelerating the rate at which microorganisms use NH_3_-N to synthesize MCP, resulting in a decrease in NH_3_-N concentration and an increase in MCP (Li et al., 2023). However, when the supplementation level is too high, it will inhibit the microbial synthesis of MCP.

Freeze-dried treatment *A. taxiformis* was more effective than other treatment methods in mitigating CH_4_ emissions and had a reduced negative impact on rumen nutrient degradation. Although the High group achieved the best CH_4_ mitigation in the freeze-dried treatment, it had a significant adverse effect on rumen fermentation parameters. We therefore conclude that the Mid group represents the optimal balance between CH_4_ emission reduction and rumen fermentation efficiency. To further explore the mechanism of freeze-dried *A. taxiformis* in mitigating CH_4_, we selected three groups of samples (CON, Low, and Mid group) for metagenomic and metabolomics analysis.

Rumen microbiome plays a crucial role in the rumen, and regulating the structure of the rumen microbiome is a common strategy to mitigate CH_4_ emissions (Fu et al., 2023). In this experiment, the supplementation of *A. taxiformis* significantly affected bacterial diversity, with the relative abundance of *Prevotella*, *Ruminobacter*, and *Succinivibrio* increased significantly in the Mid group (Figure 2H). The increase in the relative abundance of these bacteria can alleviate rumen CH_4_ emissions by competing for H_2_ utilization (Liu et al., 2022; Fu et al., 2023; Khiaosa-Ard et al., 2023). All three genera mentioned above can ferment carbohydrates in the rumen to produce succinate (Russell and Rychlik, 2001), which is subsequently converted into propionate through fermentation by *Selenomonas* (Van Gylswyk, 1995). The relative abundance of *Selenomonas* also significantly increased in the Mid group, although this genus accounted for a relatively low abundance in this experiment. This succinate pathway is the main pathway of propionate production in the rumen (Van Gylswyk, 1995; Jeyanathan et al., 2014). This result suggests that the Mid group facilitated the propionate production pathway by affecting the relative abundance of certain bacteria compared to the CON, explaining the trend of increased propionate production in the Mid group and contributing to its strong inhibition of CH_4_ production. Additionally, a significant decrease in the relative abundance of *Ruminococcus* was observed, which functions in the rumen as degraded cellulose (Baba et al., 2019). The effect was stronger in the Mid group, explaining the observed decrease in fiber degradation. The fermentation of fiber by cellulose-degrading bacteria such as *Ruminococcus* produces by-products including acetate and H_2_ (Palakawong Na Ayudthaya et al., 2018). Reducing acetate production similarly contributes to CH_4_ reduction (Jayanegara et al., 2014). At the bacterial species level, the relative abundance of *Clostridia_bacterium* and *Oscillospiraceae_bacterium*, both belonging to the Firmicutes, was significantly reduced in the Mid group (Supplementary Figure S2). Firmicutes play an important role in the degradation of carbohydrates such as starch, cellulose, and hemicellulose (Ahmad et al., 2020). The reduction in their relative abundance could also explain the decreased fiber degradation and acetate concentration in the Mid group. After supplementation of freeze-dried *A. taxiformis*, the effect on bacterial community structure with increasing levels altered the pattern of rumen VFA fermentation, promoting propionate production and inhibiting acetate production. This leads to a lower A:P ratio, which promotes the transfer of H_2_ to the propionate pathway and reduces the amount of H_2_ available for the methanogenic pathway, thereby mitigating CH_4_ emissions.

It is well known that *Methanobrevibacter* is the dominant archaeal species in the rumen, accounting for approximately 61%–74% of the total archaeal community, and is responsible for the production of CH_4_ (Choi et al., 2022; Pitta et al., 2022). This study observed a significant reduction in richness and increased diversity of archaeal community species in the Mid group compared to the CON and Low groups (Figure 3H). The relative abundance of *Methanobrevibacter* was significantly lower in the Low and Mid groups, with a more substantial reduction observed in the Mid group. Lowing the relative abundance of *Methanobrevibacter* is typical of CH_4_ inhibition. Previous studies have shown that a decreased in the relative abundance of *Methanobrevibacter* is directly responsible for the decreased in rumen CH_4_ production (Roque et al., 2019; Künzel et al., 2022; Jia et al., 2023). Additionally, the relative abundances of *Methanosphaera*, *Methanomicrobium*, and *Methanocorpusculum* were significantly higher in the Mid group. Both *Methanobrevibacter* and *Methanosphaera* are major methanogenic archaea in the rumen of ruminants. However, the relative abundance of *Methanobrevibacter* is positively correlated with CH_4_ production, whereas *Methanosphaera* is negatively correlated with methane production (Fricke et al., 2006; Cunha et al., 2019; Jia et al., 2023). This is because *Methanobrevibacter* utilizes the hydrogenotrophic pathway (M00567) consume 1 mol of CO_2_ to produce 1 mol of CH_4_, whereas *Methanosphaera* operates through methylotrophic pathway (M00356) consume 4 mol of methanol to produce 3 mol of CH_4_ (Cunha et al., 2019; Jia et al., 2023). Therefore, when the carbon source from rumen fermentation remains constant, the higher relative abundance of *Methanosphaera* results in less CH_4_ production. The results of this study indicated that the trends in the relative abundances of these two archaea might be the main reasons for the 98.53% CH_4_ reduction observed in the Mid group.

Overall, supplementation with freeze-dried *A. taxiformis* at 5% could indirectly mitigate CH_4_ emissions by affecting the bacterial community to alter the fermentation pattern of VFAs and reduce H_2_ transfer to the methanogenic pathway. Furthermore, it directly inhibited CH_4_ production in the rumen by altering the relative abundance of archaeal communities, particularly methanogenic archaea. Changes in the microbial community induced alterations in rumen fermentation patterns and functions, which strongly reduced rumen CH_4_ emissions.

Metagenomics analysis also revealed significant alterations in the KEGG pathways of the Mid group compared to the CON and Low groups. Specifically, the pathways for methane metabolism (ko00680) and carbon metabolism (ko01200) pathways were significantly suppressed, whereas the propionate metabolism pathway (ko 00640) was markedly enriched. These findings align with our experimental result, where a significant reduction in CH_4_ was observed. In the ko00680, we identified differences in the abundance of four modules: M00357 (acetate ≥ CH_4_), M00567 (CO2 ≥ CH_4_), M00563 (methylamine/dimethylamine/trimethylamine ≥ CH_4_), and M00356 (methanol ≥ CH_4_). Methanogenesis can be categorized into three pathways based on the substrate: hydrogenotrophic (M00567), aceticlastic (M00357), and methylotrophic (M00563 and M00356) (Lyu et al., 2018). In module M00357, the increased abundance of K00925 (acetate kinase [EC:2.7.2.1]) and K00625 (phosphate acetyltransferase [EC:2.3.1.8]) promoted the conversion of acetate to Acetyl-CoA (Latimer and Ferry, 1993). Changes in microbial abundance led to reduced acetate production and enhanced acetate depletion in the methanogenic pathway, contributing to a 22.66% decrease in acetate concentration in the freeze-dried Mid group compared to the CON. Additionally, the reduced abundance of M00567 in this study led to a reduction in K11261 (formylmethanofuran dehydrogenase subunit E [EC:1.2.7.12]) and K00205 (4Fe-4S ferredoxin) abundance, thus inhibiting the step in the conversion of substrate CO_2_ to Foemyl-MFR in the hydrogenotrophic pathway (Vorholt et al., 1996; Wagner et al., 2016). In the Mid group, M00563 and M00356, representing methylotrophic CH_4_-producing modules, showed significantly lower abundance, thus reducing CH_4_ production via this pathway (Figures 5C,D).

In the freeze-dried treatment of Mid group, supplementation with *A. taxiformis* reduced the abundance of K00399 (methyl-coenzyme M reductase alpha subunit [EC:2.8.4.1]). It inhibited the final reaction step of CH_4_ generation in the rumen. All three methanogenic pathways are catalyzed by MCR, a key enzyme in the final step of methane formation common to all methanogenic pathways (Lyu et al., 2018; Thauer, 2019). During this reaction, K00399 mediates the reduction of MCR by the thiol, coenzyme B (HS-CoB) to form a reductively form the heterodisulfide (CoM-SS-CoB) and CH_4_ (Lyu et al., 2018). Furthermore, CoM-SS-CoB reductive regeneration to HS-CoB and coenzyme M (HS-CoM) (Hedderich and Thauer, 1988). Previous studies have shown that the redox potential Eo′ of the CoM-S-S-CoB/HS-CoM + HS-CoB pairing is −140 mV under standard conditions (Tietze et al., 2003). Moreover, the MCR is active only when the redox potential of the CoM-S-S-CoB/HS-CoM + HS-CoB pair is well below the standard redox potential of −140 mV (Thauer, 2019). The significant increase in abundance at K03388 (heterodisulfide reductase subunit A2 [EC:1.8.98.4]) and K03389 (heterodisulfide reductase subunit B2 [EC:1.8.98.4]) facilitated the process of CoM-S-S-CoB reduction to generate HS-CoM and HS-CoB, resulting in an elevated redox potential Eo′ for the CoM-S-S-CoB/HS-CoM + HS-CoB pair, which decreased MCR activity, thereby inhibiting CH_4_ emission.

Metabolomics allows for analyzing rumen fermentation metabolites *in vitro* and identifying biomarkers that reflect the physiological status of ruminants (Fontanesi, 2016). In the current study, the Proximal Tubule Bicarbonate Reclamation metabolic pathway was significantly enriched in the Mid group compared to the CON. As shown in Supplementary Table S2, the major differential metabolites within this pathway were L-glutamate, ketoglutarate, and malate. L-glutamate produces ketoglutarate, which enters the tricarboxylic acid cycle (TCA) (Hinca et al., 2021). The Mid group also significantly enriched the TCA metabolic pathway, with ketoglutarate, isocitrate, and malate as the main differential metabolites. The accumulation of these three TCA cycle upstream metabolites suggests a potential inhibited of dehydrogenation reaction in the TCA process (Choi et al., 2021), this inhibition would limit H_2_ and CO_2_ during sugar metabolism, thereby reducing substrate for CH_4_ production. We hypothesize that the bioactive compounds of *A. taxiformis* may target key TCA enzymes, though direct mechanistic evidence requires future *in vitro* enzymology studies.

Furthermore, we observed that the highest enrichment in the Mid group was in the Tryptophan metabolic. Within this pathway, melatonin was identified as a differential metabolite. Besides its well-known roles in anti-inflammatory, sleep-promoting, mood-improving, reproductive, and immune-enhancing activities, melatonin’s antioxidant function is widely recognized (Tan et al., 2015). Several studies have shown that melatonin can regulate the activity of intestinal microbes or their metabolites and improve intestinal physiology (Rahman et al., 2005; Ren et al., 2018; Fu et al., 2023). Recent studies have shown that melatonin mitigates about 50% of CH_4_ emissions in the rumen of dairy cows by decreasing the relative abundance and production of CH_4_ substrates by methanogenic archaea (Fu et al., 2023). In the present study, melatonin was significantly enriched in both the Tryptophan Metabolic and Circadian Entrainment pathways. The elevated expression of melatonin may play a role in reducing methane emissions.

## 5 Conclusion

**Figure 7:**
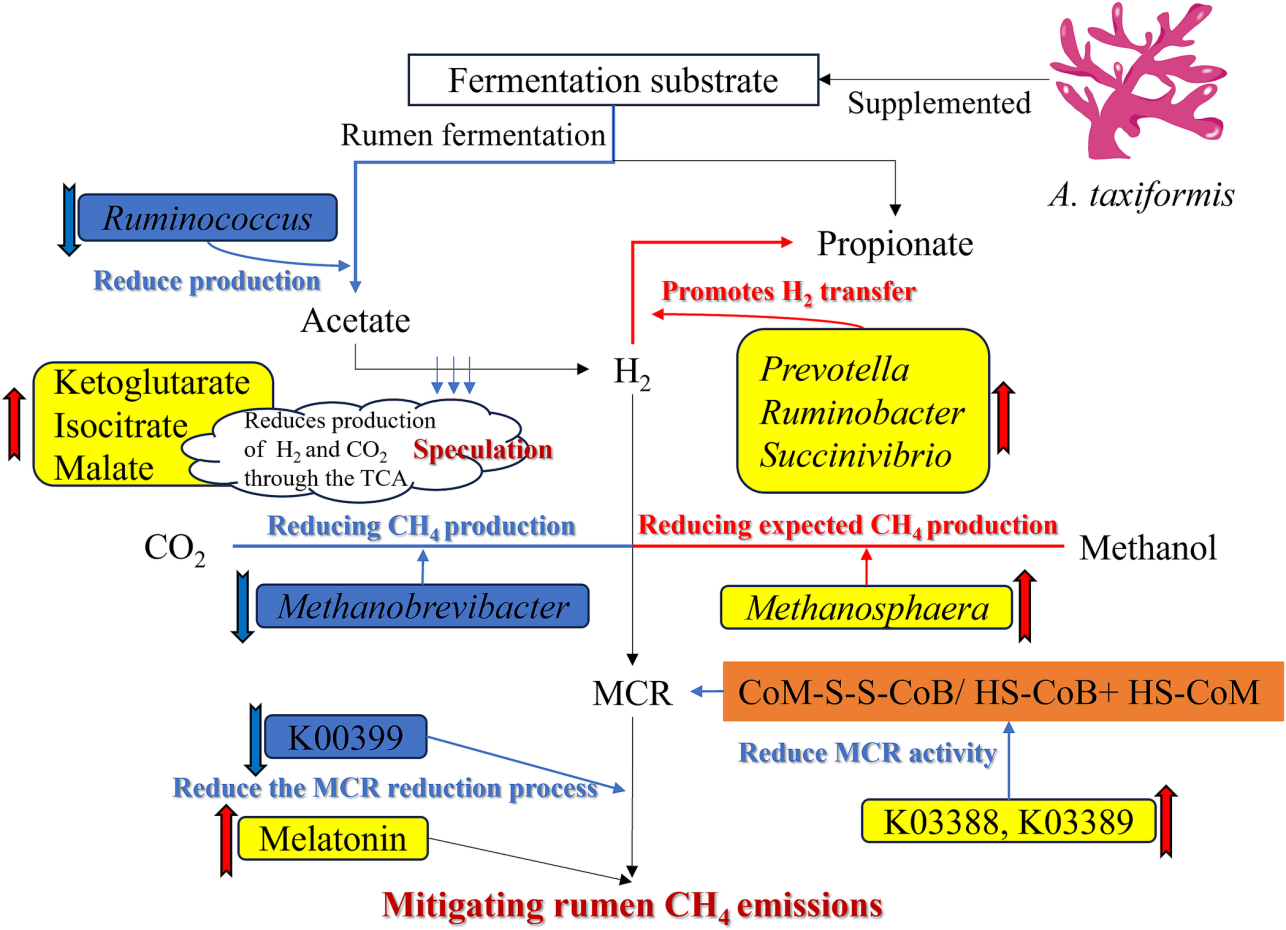
Mechanism of supplementation of *Asparagopsis taxiformis* to alleviate methane emission in the *in vitro* rumen fermentation.

Supplementation with 5% freeze-dried *A. taxiformis* significantly mitigated *in vitro* rumen fermentation CH₄ emissions, reducing CH₄ by 98.53%. The potential mechanism of *A. taxiformis* on the rumen contributed was drawn in Figure 7 to this substantial reduction in CH_₄_ emissions. Thereof, the supplementation of *A. taxiformis* increased the abundance of *Prevotella*, *Ruminobacter*, and *Succinivibrio*, and reduced the abundance of *Ruminococcus*, resulting in a decrease in the net H_₂_ balance and indirectly mitigated CH_₄_ emissions. Supplementation also directly influenced methanogenesis pathway by altering the abundance of key archaea. Specifically, the upregulation of K03388 (heterodisulfide reductase subunit A2) and K03389 (heterodisulfide reductase subunit B2) facilitated the reduction of CoM-SS-CoB to HS-CoB and HS-CoM, inhibiting MCR enzyme activity. Concurrently, the downregulation of K00399 (methyl-coenzyme M reductase alpha subunit) further inhibited MCR reduction to generate CH_₄_, collectively suppressing the final step of CH_₄_ production in the rumen and reducing overall CH_₄_ emissions. Moreover, melatonin expression was significantly upregulated, reducing CH_₄_ emissions through its dual role as an antioxidant and microbial modulator. These findings suggest that *A. taxiformis* supplementation may offer a promising and biologically plausible approach to sustainable ruminant production. Further animal studies are warranted to comprehensively evaluate the long-term CH_₄_ reduction potential of *A. taxiformis*, its effects on animal health and performance, as well as its impacts on milk and meat quality, and economic feasibility.

## Data Availability

The datasets presented in this study can be found in online repositories. The names of the repository/repositories and accession number(s) can be found at: https://www.ncbi.nlm.nih.gov/genbank/, PRJNA1148834.

## Footnotes

^1^ www.illumina.com

^2^ http://www.hmdb.ca/

^3^ https://metlin.scripps.edu/
