# Supplementary material for: Asparagopsis taxiformis mitigates ruminant methane emissions via microbial modulation and inhibition of methyl-coenzyme M reductase
Source: Front Microbiol. 2025 Apr 25;16:1586456. doi: 10.3389/fmicb.2025.1586456 (PMC12061954; doi:10.3389/fmicb.2025.1586456)
Supplement: Supplementary file 1 [file Data_Sheet_1.ZIP › Supplementary Material/Supplementary Fig.docx]

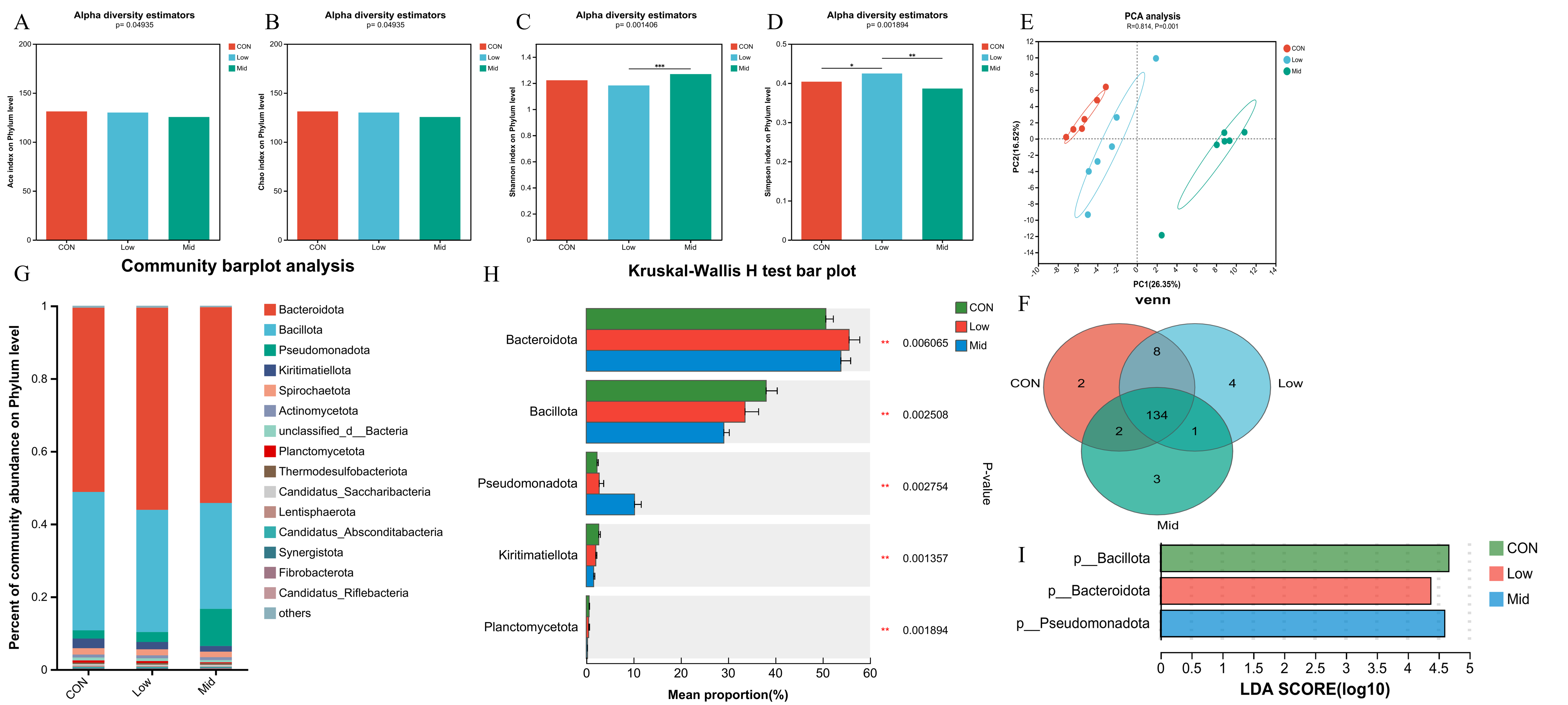


Fig S1. Effect of different treatments and supplementation levels of *A. taxiformis* on rumen bacterial composition in the *in vitro* rumen fermentation. **A** Ace index on phylum level. **B** Chao index on phylum level. **C** Shannon index on phylum level. **D** Simpson index on phylum level. **E** Beta diversity. **F** Venn diagram on phylum level. **G** Relative abundances of the 15 most abundant phylum-level across all three groups. **H** Differences in bacterial phylum levels by metagenomics sequencing. **I** The LDA values ​​of different species among the three groups on phylum level (LDA>4). CON, control group; Low, CON plus 2% *A. taxiformis*; Mid, CON plus 5% *A. taxiformis*; High, CON plus 10% *A. taxiformis*. *<0.05, **<0.01, ***<0.001


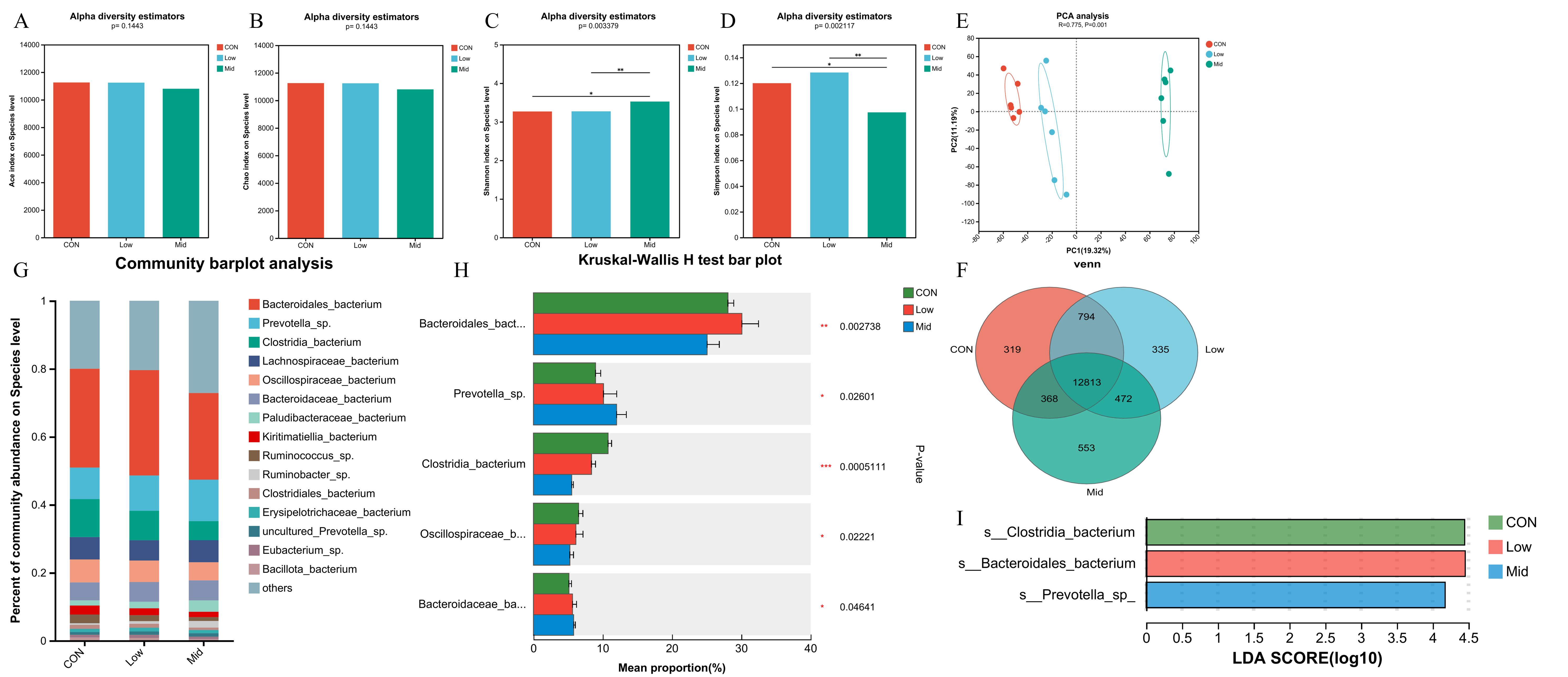


Fig S2. Effect of different treatments and supplementation levels of *A. taxiformis* on rumen bacterial composition in the in vitro rumen fermentation. **A** Ace index on species level. **B** Chao index on species level. **C** Shannon index on species level. **D** Simpson index on species level. **E** Beta diversity. **F** Venn diagram on species level. **G** Relative abundances of the 15 most abundant species-level across all three groups. **H** Differences in bacterial species levels by metagenomics sequencing. **I** The LDA values ​​of different species among the three groups on species level (LDA>4). CON, control group; Low, CON plus 2% *A. taxiformis*; Mid, CON plus 5% *A. taxiformis*; High, CON plus 10% *A. taxiformis*. *<0.05, **<0.01, ***<0.001


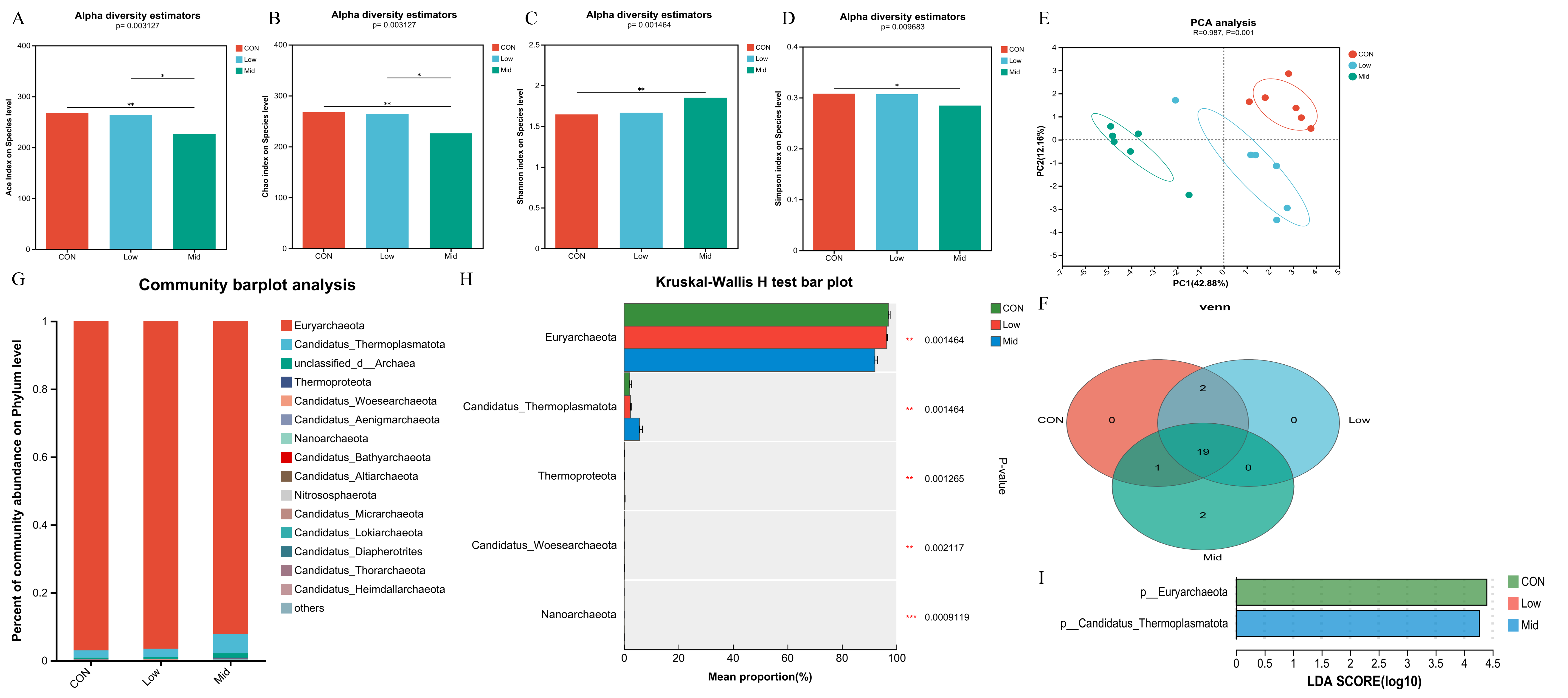


Fig S3. Effect of different treatments and supplementation levels of *A. taxiformis* on rumen archaea composition in the in vitro rumen fermentation. **A** Ace index on species level. **B** Chao index on species level. **C** Shannon index on species level. **D** Simpson index on species level. **E** Beta diversity. **F** Venn diagram on species level. **G** Relative abundances of the 15 most abundant species-level across all three groups. **H** Differences in archaea species levels by metagenomics sequencing. **I** The LDA values ​​of different species among the three groups on species level (LDA>4). CON, control group; Low, CON plus 2% *A. taxiformis*; Mid, CON plus 5% *A. taxiformis*; High, CON plus 10% *A. taxiformis*. *<0.05, **<0.01, ***<0.001


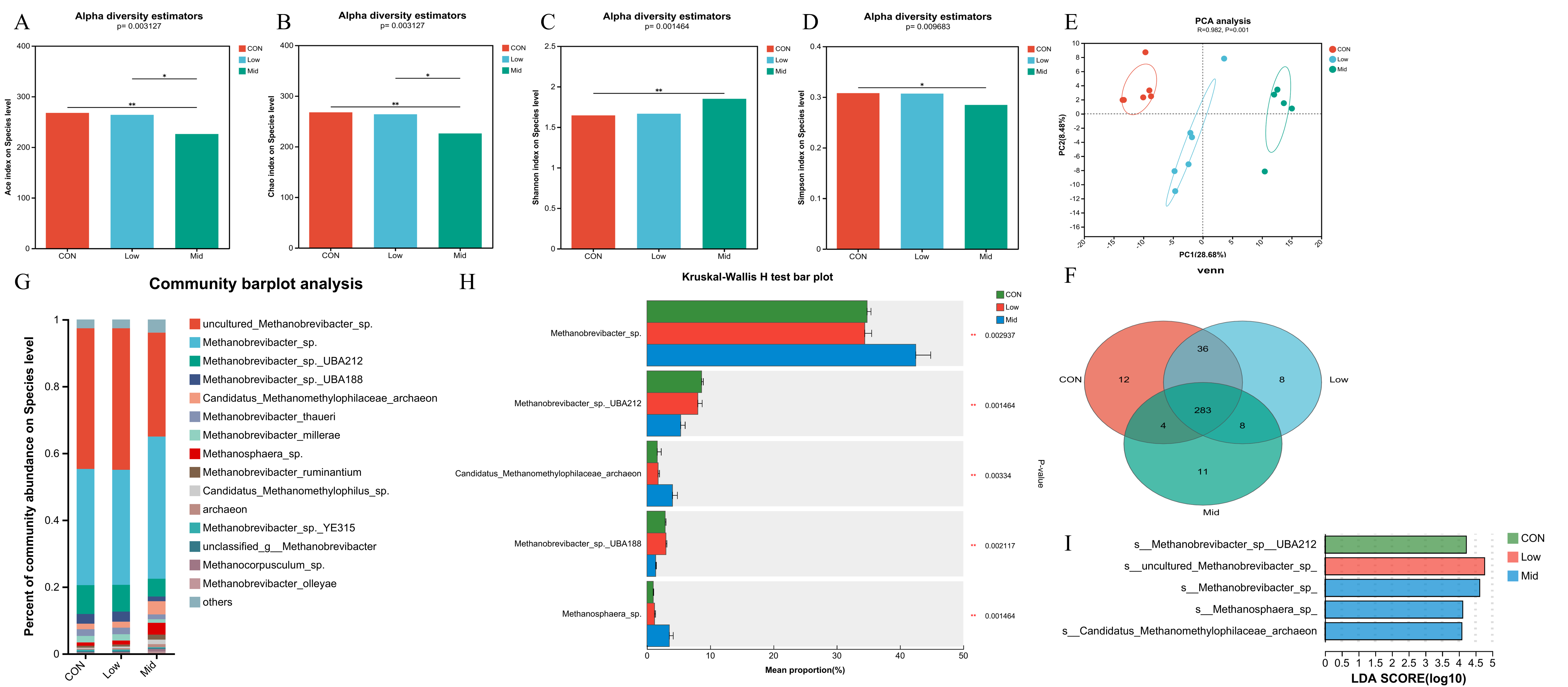


Fig S4. Effect of different treatments and supplementation levels of *A. taxiformis* on rumen archaea composition in the in vitro rumen fermentation. **A** Ace index on species level. **B** Chao index on species level. **C** Shannon index on species level. **D** Simpson index on species level. **E** Beta diversity. **F** Venn diagram on species level. **G** Relative abundances of the 15 most abundant species-level across all three groups. **H** Differences in archaea species levels by metagenomics sequencing. **I** The LDA values ​​of different species among the three groups on species level (LDA>4). CON, control group; Low, CON plus 2% *A. taxiformis*; Mid, CON plus 5% *A. taxiformis*; High, CON plus 10% *A. taxiformis*. *<0.05, **<0.01, ***<0.001
