# Supplementary material for: Asparagopsis taxiformis mitigates ruminant methane emissions via microbial modulation and inhibition of methyl-coenzyme M reductase
Source: Front Microbiol. 2025 Apr 25;16:1586456. doi: 10.3389/fmicb.2025.1586456 (PMC12061954; doi:10.3389/fmicb.2025.1586456)
Supplement: Supplementary file 1 [file Data_Sheet_1.ZIP › Supplementary Material/Table S1.docx]

**Table S1**. The cumulative total gas, gas composition of two treatments, and different supplementation levels after *in vitro* rumen fermentation for 48 h

| Parameter | CON | Low | Mid | High | *P* |
| --- | --- | --- | --- | --- | --- |
| **Dried** | | | | | |
| TGP mL | 146.60±6.03^A^ | 142.44±8.34^A^ | 128.01±11.03^A^ | 104.43±22.07^B^ | <0.001 |
| H_2_ mL | 0.03±0.01^B^ | 0.04±0.01^B^ | 0.43±0.07^B^ | 1.80±0.73^A^ | <0.001 |
| CH_4_ mL | 9.18±0.44^A^ | 9.88±0.67^A^ | 7.16±0.67^B^ | 2.53±0.89^C^ | <0.001 |
| CO_2_ mL | 67.91±2.33 | 65.41±3.85 | 68.72±3.20 | 67.98±6.34 | 0.55 |
| TGP mL /g DM | 293.02±12.02^A^ | 279.08±16.37^A^ | 243.63±21.02^B^ | 189.72±40.10^C^ | <0.001 |
| H_2_ mL /g DM | 0.06±0.02^B^ | 0.09±0.01^B^ | 0.83±0.13^B^ | 3.27±1.34^A^ | <0.001 |
| CH_4_ mL /g DM | 18.35±0.89^A^ | 19.37±1.32^A^ | 13.63±1.27^B^ | 4.60±1.62^C^ | <0.001 |
| CO_2_ mL /g DM | 135.74±4.65 | 128.16±7.55 | 130.79±6.07 | 123.51±11.52 | 0.08 |
| **Freeze-dried** | | | | | |
| TGP mL | 146.60±6.03^A^ | 130.09±2.51^B^ | 116.755.62^C^ | 124.90±4.85^B^ | <0.001 |
| H_2_ mL | 0.03±0.01^C^ | 0.41±0.10^C^ | 3.26±0.33^B^ | 3.96±0.77^A^ | <0.001 |
| CH_4_ mL | 9.18±0.44^A^ | 7.11±0.18^B^ | 0.17±0.05^C^ | 0.07±0.01^C^ | <0.001 |
| CO_2_ mL | 67.91±2.33 | 71.04±1.35 | 70.92±1.79 | 69.89±2.96 | 0.08 |
| TGP mL /g DM | 293.03±12.02^A^ | 254.87±4.89^B^ | 222.17±10.72^C^ | 226.92±8.84^C^ | <0.001 |
| H_2_ mL /g DM | 0.06±0.02^C^ | 0.81±0.21^C^ | 6.21±0.63^B^ | 7.21±1.40^A^ | <0.001 |
| CH_4_ mL /g DM | 18.35±0.89^A^ | 13.93±0.36^B^ | 0.33±0.10^C^ | 0.14±0.01^C^ | <0.001 |
| CO_2_ mL /g DM | 135.74±4.65^A^ | 139.19±2.63^A^ | 134.96±3.39^A^ | 126.98±5.36^B^ | <0.001 |

CON, control group; Low, CON plus 2% *A. taxiformis*; Mid, CON plus 5% *A. taxiformis*; High, CON plus 10% *A. taxiformis*.

^ab^ Means bearing different superscripts in the same row differ significantly (P<0.05).

^AB^ Means bearing different superscripts in the same row differ significantly (P<0.01).
